# Green biosynthesis of rare DHA-phospholipids by lipase-catalyzed transesterification with edible algal oil in solvent-free system and catalytic mechanism study

**DOI:** 10.3389/fbioe.2023.1158348

**Published:** 2023-03-31

**Authors:** Tiantian Zhang, Binglin Li, Zhulin Wang, Dan Hu, Xiaoli Zhang, Binxia Zhao, Jiao Wang

**Affiliations:** ^1^ College of Food Science and Engineering, Northwest University, Xi’an, China; ^2^ College of Chemical Engineering, Northwest University, Xi’an, China; ^3^ Biochemistry Center (BZH), Heidelberg University, Heidelberg, Germany; ^4^ BioQuant, Heidelberg University, Heidelberg, Germany

**Keywords:** DHA-PLs, transesterification, edible oil system, molecular dynamics, molecular docking

## Abstract

Docosahexaenoic acid (DHA)-enriched phosphatidylcholine (PC) has received significant scientific attention due to the health benefits in food and pharmaceutical products. In this work, the edible algal oil rich in DHA-triacylglycerol (DHA-TAG) without pretreatment was first used as the DHA donor for the transesterification of phospholipids (PLs) to prepare three kinds of rare PLs, including DHA-PC, DHA-phosphatidylethanolamine (DHA-PE), and DHA-phosphatidylserine (DHA-PS). Here, 153 protein structures of triacylglycerol lipase (EC 3.1.1.3) were virtually screened and evaluated by transesterification. PLA1 was the best candidate due to a higher DHA incorporation. Results showed that the transesterification of PC with DHA-TAG at 45°C and 0.7% water content (without additional water addition) could produce DHA-PC with 39.1% DHA incorporation at 30 min. The different DHA donors, including forms of fatty acid, methyl ester, and triglycerides, were compared. Molecular dynamics (MD) was used to illustrate the catalytic mechanism at the molecular level containing the diffusions of substrates, the structure-activity relationship of PLA1, and the effect of water content.

## 1 Introduction

Docosahexaenoic acid-enriched phospholipids (DHA-PLs) have received significant scientific attention due to their health benefits in functional food and pharmaceutical products. Current research suggested that DHA-PLs have potential benefits in improving neurocognitive disorders by regulating oxidative stress and inflammatory responses (Chouinard-Watkins et al., 2019; Hachem et al., 2020). DHA-PLs can cross the blood-brain barrier (BBB) and participate in brain biochemical reactions (Ahmmed et al., 2020). Hence, DHA-PLs exhibited higher bioavailability and antioxidation than DHA-TAG such as fish oil and algal oil (Ahmmed et al., 2020; Hachem et al., 2020; Zhang et al., 2020).

DHA-PLs can be obtained by a variety of strategies--physical extraction from natural products, microbial fermentation, chemical synthesis, and enzymatic synthesis. Marine sources such as algae, krill, shrimp, and mussels are the most important and available natural sources for DHA-PLs (Kris-Etherton et al., 2003; Ahmmed et al., 2020). However, the difficulties that existed in the process of physical extraction are the limited supply of raw materials and the safety hazards such as heavy metal contamination due to marine pollution. This is detrimental to the application of DHA-PLs in the food and pharmaceutical industries. O Hidetoshi et al. disclosed a method to produce DHA-PLs using a microorganism in a simpler manner (Hidetoshi et al., 2012). The phospholipids obtained only accounted for about 10% of the total lipids. The strain was not optimized with the low selectivity for fatty acids. The product composition was complex. Therefore, the microbial fermentation was still in an immature stage. Phosphatidylcholine (PC) bearing DHA at the 2-positions was synthesized chemically by Naomichi Baba et al. (Baba et al., 2001). Ann-Marie Lyberg et al. have incorporated DHA in LPC with a chemical method using EDCI as a coupling agent and DMAP as a catalyst (Haraldsson et al., 2000; Lyberg et al., 2005). Chemical synthesis had a higher reaction rate and yield, but the reaction process was complex and the reaction conditions were harsh, reducing the production safety.

The widely used strategies to incorporate DHA into PLs are enzymatic transesterification or esterification. The enzymatic reaction presents advantages related to mild reaction conditions, high selectivity, and high production safety (He et al., 2022). Generally, lipase-mediated transesterification of PC with DHA-rich ester (DHA-ethyl ester (DHA-EE) and DHA-TAG) and acidolysis of PC with DHA (free fatty acids) are used to synthesize DHA-PLs. Several studies have been published on the production of DHA-PLs by lipase-catalyzed methods (shown in Table 1). DHA and DHA-EE prepared from fish oil were the most used acyl donors. Compared with the DHA, the DHA-EE and DHA-TAG could provide better fluidity at room temperature, which benefits the reaction proceeding. Furthermore, edible DHA-TAGs (fish oil and algae oil) are good candidates to be the DHA donor without saponification or transesterification to prepare DHA or DHA-EE. However, the fish oil might not be the best candidate due to the fishy smell, low DHA content, and potential animal infectious diseases (Ansorena and Astiasarán, 2013). Algae oil is a promising and sustainable option. Algae can grow faster and can be grown in the sea, in tanks, and on land not suitable for cultivation of normal crops, meaning no competition with agricultural land. Algae culture is environmentally friendly as it uses CO_2_ for biomass growth (Kris-Etherton et al., 2003).

**TABLE 1:** Studies on the lipase-catalyzed synthesis of DHA-PLs in the past decade.

Substrates	Method	System	Enzyme	Product form (maximum incorporation)	Reference
Soybean lecithin with EPA/DHA-MEs	Transesterification	Mg^2+^/hexane	Lipozyme^®^ RM IM	EPA + DHA incorporated lecithin, 12.30%^a^	Marsaoui et al. (2013)
DHA/EPA-EEs with PC	Transesterification	Solvent-free	Immobilized PLA1	EPA + DHA incorporated PC, 30.7%^a^	Li et al. (2014)
Soy-PL and PUFA-EEs	Transesterification	Hexane	Lipase OF from *C. rugosa*	PUFA-PLs, (47.1 ± 2.1)wt%^b^	Yamamoto et al. (2014)
PUFA (from fish oil) and PC	Transesterification (acidolysis)	Solvent-free	Immobilized PLA1	PUFA-PC, 57.4 mol%^b^	Zhao et al. (2014)
Soybean PL with EPA/DHA -MEs	Transesterification	Mg^2+^/urea, solvent-free	Lipozyme^®^ RM IM	EPA + DHA incorporated PL, 45.7%^a^	Marsaoui et al. (2015)
PC (from Antarctic krill) and FA (from fish oil)	Transesterification (acidolysis)	Supercritical carbon dioxide (SCCO_2_)	Immobilized PLA1	DHA-PC, 59.0 mol%	Xi et al. (2016)
PS and DHA	Transesterification (acidolysis)	Glycerol	Recombinant porcine pancreas PLA2	2-DHA-PS, -	Liu et al. (2016)
PC and DHA/EPA-EEs	Transesterification	Solvent-free (vacuum)	Immobilized PLA1	DHA/EPA-PC, 30.31%	Li et al. (2016)
Soybean and DHA (from alga oil)	Transesterification (acidolysis)	Reverse micelles	PLA1	DHA-PC, 20.90%	Chen et al. (2017b)
GPC and PUFA (from fish oil)	Esterification	Solvent-free	Lipozyme^®^ RM IM	PUFA-LPC, -	Liu et al. (2017)
GPC and PUFA	Esterification	Solvent-free (vacuum)	Immobilized MAS1 Lipase	PUFA-LPC, 89.36%^b^	Wang et al. (2020)
GPS and PUFA	Esterification	Solvent-free (vacuum)	Lipozyme^®^ RM IM	PUFA-LPS, 71.63 mol%	Zhang et al. (2022)

^a^ Refers to the sum incorporation of DHA, and EPA.

^b^ Refers to the sum incorporation of PUFA.

Abbreviations: EPA, eicosapentaenoic acid; PUFA, polyunsaturated fatty acid; MEs, methyl esters; EEs, ethyl esters; PC, phosphatidylcholine; GPC, sn-glycero-3-glycerylphosphorylcholine; LPC, lysophosphatidylcholine; GPS, sn-glycero-3-phosphatidylserine.

This paper demonstrated a reaction system that DHA-PLs were prepared by lipase-mediated transesterification of PLs with edible DHA-TAG from algae oil in a solvent-free system. The molecular docking virtually screened 158 protein structures of triacylglycerol lipase (EC 3.1.1.3) for transesterification *in silico*. Screened enzymes were further evaluated by transesterification to prepare DHA-PLs. The reaction parameters containing the reaction temperature and water content were evaluated. In addition, the effects of different acyl donors [DHA, DHA-methyl ester (DHA-ME), and DHA-TAG] and different kinds of phospholipid [PC, phosphatidyl ethanolamine (PE), and phosphatidylserine (PS)] on the preparation of DHA-PLs were systematically investigated in the present work. Finally, the molecular dynamics (MD) simulation was employed to quantitatively reconstruct the whole enzymatic processes, analyze their kinetic behaviors, illustrate catalytic mechanism, and explain our experimental phenomenon at the molecular level.

## 2 Materials and methods

### 2.1 Materials

Phosphatidylcholine (PC>98%), phosphatidyl ethanolamine (PE>80%) and phosphatidylserine (PS>50%) was purchased from Merya’s lecithin co., ltd. (Beijing, China). Refined DHA-rich algal oil from *Schizochytrium* sp. (DHA ≥60%) was kindly provided by Qingdao Xunon Bioengineering Co., Ltd. (Qingdao, China). DHA and DHA-ME were prepared from algal oil. The 37 kinds of fatty acid methyl ester standards were purchased from Sigma-Aldrich Co., Ltd. (Norcross, GA). Lipozyme^®^CALB L (from *Candida antarctica*, CALB), Lipozyme^®^ TL 100 L (from *Thermomyces lanuginosus*, TL100), Novocor^®^AD L (from *Candida antarctica*, ADL) Novozym^®^51,032 (from *Aspergillus oryzae*, 51,032), Resinase^®^HT (from *Aspergillus oryzae*, HT), Lecitase^®^ Ultra (from *Thermomyces lanuginosus/Fusarium oxysporum*, PLA_1_), Novozym^®^435 (from *Candida antarctica*, 435), and Lipozyme^®^TL IM (from *Thermomyces lanuginosus*, IM) were purchased from Novozymes Biotechnology Co., LTD. (Tianjin, China). The thin-layer chromatography plates coated with silica gel G were purchased from Qingdao Ocean Chemical Co., Ltd. (Qingdao, China). Other chemicals and solvents applied were purchased from Sinopharm Chemical Reagent Co., Ltd. (Shanghai, China) with chromatographic and analytical grade.

### 2.2 Virtual screening of enzymes for transesterification

Molecular docking, including docking and reverse docking, is a fast and efficient computational method to predict the bioactive compounds to a specific protein or reversely predict the target proteins for one bioactive compound (Chen et al., 2017). The docking and reverse docking procedures were performed as follows:

(1) Establishment of the enzyme Protein Data Bank (PDB) database: all 158 protein structures of triacylglycerol lipase (EC 3.1.1.3) were collected from the RCSB data bank (www.rcsb.org) and pretreated with PyMOL to remove the water molecules, heteroatoms, ions, and original ligands (Schrodinger, 2015). Each geometric center of the protein was measured according to its catalytic triad residues. The size of the grid box was set of 22.5 × 22.5 × 22.5 Å^3^;
(2) Ligands’ preparation: DHA-TAG is the most important ligand because it is the most abundant component of algal oil. Lipase is a kind of hydrolase; the occurrence of hydrolysis was inevitable in the present system. The hydrolysates were DHA-diacylglycerol (2,3-DHA-DAG) and DHA-monoacylglycerol (2-DHA-MAG) due to a large steric hindrance of sn-2 position. Therefore, 2,3-DHA-DAG and 2-DHA-MAG may also act as acyl donors for DHA. For phospholipids, C16:0 and C18: 2 were the most abundant fatty acids. As a result, 2,3-dipalmitoyl phosphatidylcholine (DPPC) and 2,3-dilinoleoyl phosphatidylcholine (DUPC) were suitable representatives of phospholipids.

The structural files for esters of DHA (DHA-TAG, DHA-DAG, DHA-MAG), free fatty acids (DHA, C18:2, C16:0), and phospholipids of DPPC and DUPC were downloaded from ZINC 15 and CHARM-GUI database (Jo et al., 2009; Sterling and Irwin, 2015); (3) Docking: all PDB files were converted into PDBQT files by AutoDockTools with the version of 1.5.6 (Morris et al., 2009); molecular docking was carried out by AutoDock Vina with the version of 1.1.2 by the Broyden–Fletcher–Goldfarb–Shanno (BFGS) method (Trott and Arthur, 2009; Eberhardt et al., 2021); other parameters were used as defaults. Python with the version of 3.7 was used as the working language for all software in this work.

### 2.3 Lipase-mediated transesterification of PLs with edible DHA-TAG from algae oil in a solvent-free system

PLs (20 mg) were mixed with 1.0 g of algae oil in screw-capped Erlenmeyer flasks (5 mL capacity) under ultrasonic vibration until completely dissolved. Lipase (7 mg for liquid lipases and 20 mg for immobilized lipases) was added to start the enzymatic reaction after preheating the mixture to the reaction temperature and maintaining for 5 min. The mixture was incubated at various temperatures (40, 45, 50, 55, and 60°C) in an incubator at 500 r/min. Individual samples (50 µL) were withdrawn at selected times and analyzed.

### 2.4 Separation and methyl esterification of PL and LPL

The same volume of methanol (50 µL) was added to the individual samples to extract PLs from the mixture. DHA-PLs and DHA-lyso-phospholipids (DHA-LPLs) were confirmed on thin-layer chromatography (TLC) using a solvent system of chloroform/methanol/water (13:5:0.8, v/v/v). The bands were sprayed with 0.2% 2,7-dichlorofluorescein in methanol and visualized under ultraviolet (UV) light. Then the bands of DHA-PLs and DHA-LPLs were scraped and eluted with a small volume of chloroform-methanol (2:1, v/v). Then methylated to fatty acid methyl ester (FAME) according to American Oil Chemists Society (AOCS) standard method 996.01 and carried out for gas chromatography (GC) analysis.

PLs, LPLs, or glyceride were mixed with 0.5 mL of 2 M potassium hydroxide methanol solution under vigorous shaking for 1 min. The obtained solution was further stood for 5 min. Chromatographic isooctane (1 mL) was used to extract FAMEs. The isooctane phase was collected; anhydrous sodium sulfate was added to remove water. The upper liquid was collected by centrifugation (9,000×*g*, 5 min) to analyze the fatty acid compositions by GC. The yields of PC and LPC in bands of TLC can be calculated according to the total FA amount. One mole of PC methylated 2 mol of FAME, and 1 mol of LPC methylated 1 mol of FAME.

### 2.5 GC analysis

A gas chromatograph (GC 2030, Shimadzu, Japan) equipped with a flame-ionization detector was used to analyze the FAMEs contents. The column was SH-Rtx-wax (30 m × 0.25 mm×0.25 um). The oven temperature was held at 165°C for 1 min, then increased to 210°C with a rate of 6.5°C/min, increased to 220°C at a rate of 1.5°C/min, and maintained for 1 min. The temperatures of the injector and detector were held at 250°C and 280°C, respectively. The flow rates of N_2_, H_2_, and air were 24, 32, and 200 mL/min.

### 2.6 Dynamic simulation

To study kinetic behaviors, the systems of micro-water and anhydrous were generated by the software Packmol (Schrodinger, 2015). The two micro-units were generated in a cubic TIP3P box of 61 × 61 × 91 Å^3^. Each of them had one lipase molecule (PDB ID: 6xok) (McPherson et al., 2020), 20 of PC molecules and 200 of DHA-TAG molecules. The micro-water system had 770 water molecules, only covering on the protein surface. The catalytic triad consisted of Ser146, Asp201, and His258. MD simulation was performed by NAMD with version of 2.13 (Phillips et al., 2010). The CHARMM36 force field was performed in all cases (Huang and Mackerell, 2013). The topology files of ligands could be generated by CGenFF (Vanommeslaeghe et al., 2010). Some topology files which were not included in the standard CHARMM36 force field and cannot be auto generated were created by ourselves. The minimization consisted of 5,000 steps conjugate gradient energy minimization to relax all atoms. Then, the temperature of the system was gradually raised to 313 K in a 4 ns relaxation. Next, all MD simulations were operated for 4 times with each time scale of 240 ns under normal pressure. Other conditions were set as default values.

All experiments were performed in triplicate. Data were shown as mean value with the standard deviation (mean ± SD).

## 3 Results and discussion

### 3.1 Molecular docking for virtual screening of lipases

**FIGURE 1:**
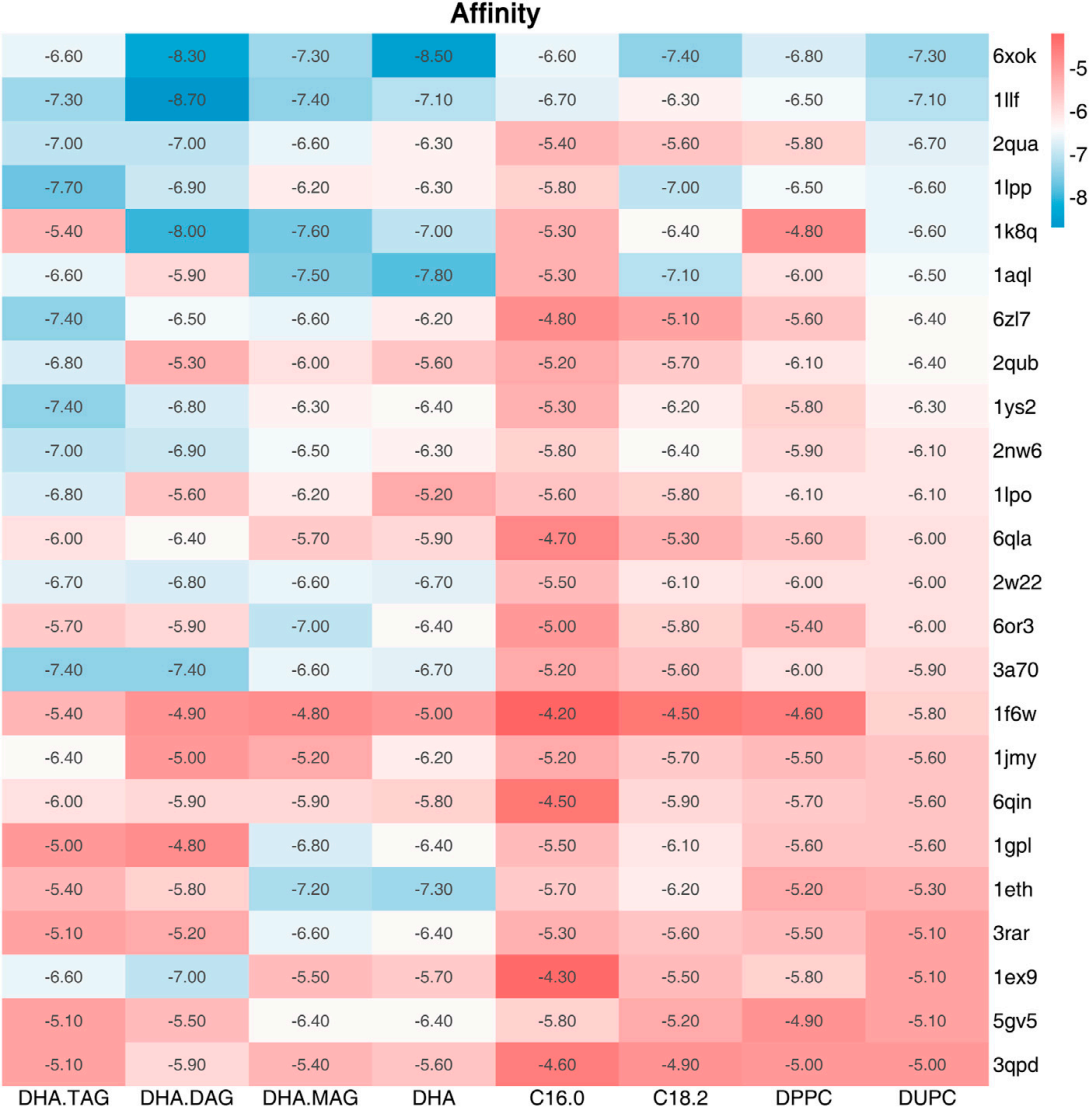
Affinity (kcal/mol) between the ligand and the screened lipases (Affinity ≤ −5.0 kcal/mol and Distance≤4.5 Å).

**FIGURE 2:**
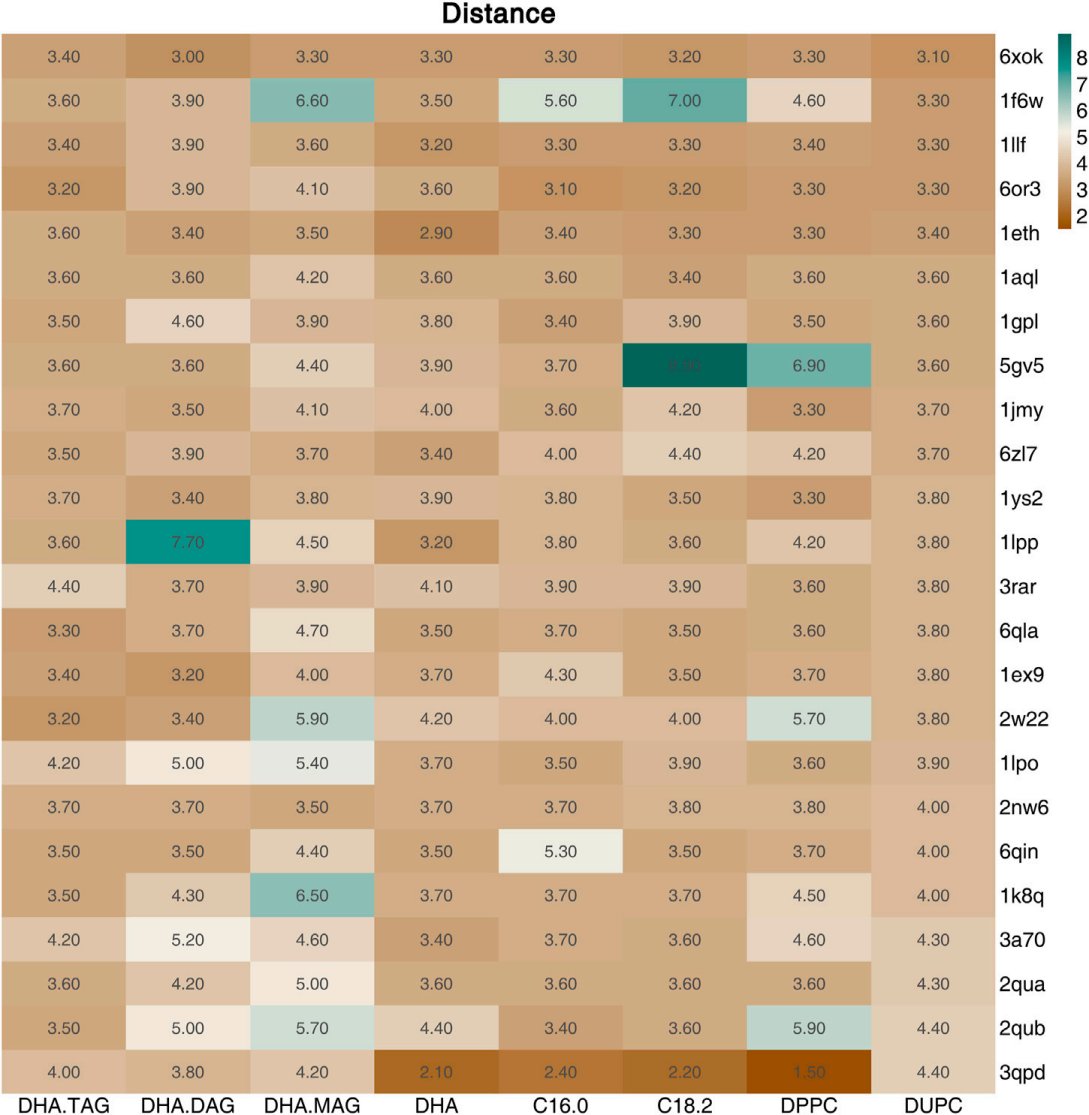
Distance (Å) between the Oγ on the Ser of the screened lipases (Affinity ≤ −5.0 kcal/mol and Distance≤4.5 Å) and the acyl carbon atom of the ligand.

The catalytic triad of lipases with reported crystal structures is composed of Ser-His-Asp/Glu (Liu et al., 2022). In the lipase-mediated transesterification of PLs with edible DHA-TAG, the Oγ atom on the Ser residue in the catalytic triad initiates the nucleophilic attack to the acyl carbon atom on the ligand (the substrates). Therefore, we performed a virtual screening of lipases basing on the critical distance between the Oγ atom of the key Ser in the catalytic triad and the acyl carbon atom of the substrate, and their affinity. Among all the docking and reverse docking results, there were 85 structures with smaller distances (<8.0 Å) and negative affinities shown in Supplementary Tables S1, S2. In addition, the two main ligands of DHA-TAG and DUPC were focused. The cutoff values of docking affinity and crucial distance were set to −5.0 kcal/mol and 4.0 Å, respectively. As shown in Table 2; Figures 1, 2, 24 potential structures were obtained and classified by their organisms. Basing on these lipase structures, eight kinds of commercial lipases were confirmed.

**TABLE 2:** The screened structures with better performances in both affinity and distance and their organisms.

PDB ID	Organism	Available commercial lipases
2w22	*Geobacillus thermocatenulatus*	-
6or3	*Thermomyces lanuginosus*	Lipozyme^®^ TL 100 L
6xok		Lecitase^®^ Ultra
		Lipozyme^®^TL IM(immobilized)
6qla	*Uncultured bacterium*	-
6zl7		
6qin		
1ex9	*Pseudomonas aeruginosa*	-
1llf	*Debaryomycetaceae* sp.	-
	*‘Limtongozyma cylindracea*	
1gpl	*Cavia porcellus*	-
1k8q	*Canis lupus familiaris*	-
2qub	*Serratia marcescens*	-
1lpp	*Diutina rugosa*	-
1lpo		
3rar		
1f6w	*Homo sapiens*	-
1jmy		
1aql	*Bos taurus*	-
1eth	*Sus scrofa*	-
2qua	*Serratia marcescens*	-
5gv5	*Moesziomyces antarcticus*	Lipozyme^®^CALB L
		Novocor^®^AD L
		Novozym^®^435 (immobilized)
1ys2	*Burkholderia cepacia*	-
2nw6		
3qpd	*Aspergillus oryzae*	Novozym^®^51,032
		Resinase^®^HT
3a70	*Pseudomonas* sp. MIS38	-

### 3.2 Evaluation of lipases by transesterification of PLs with edible DHA-TAG from algae oil in a solvent-free system

**FIGURE 3:**
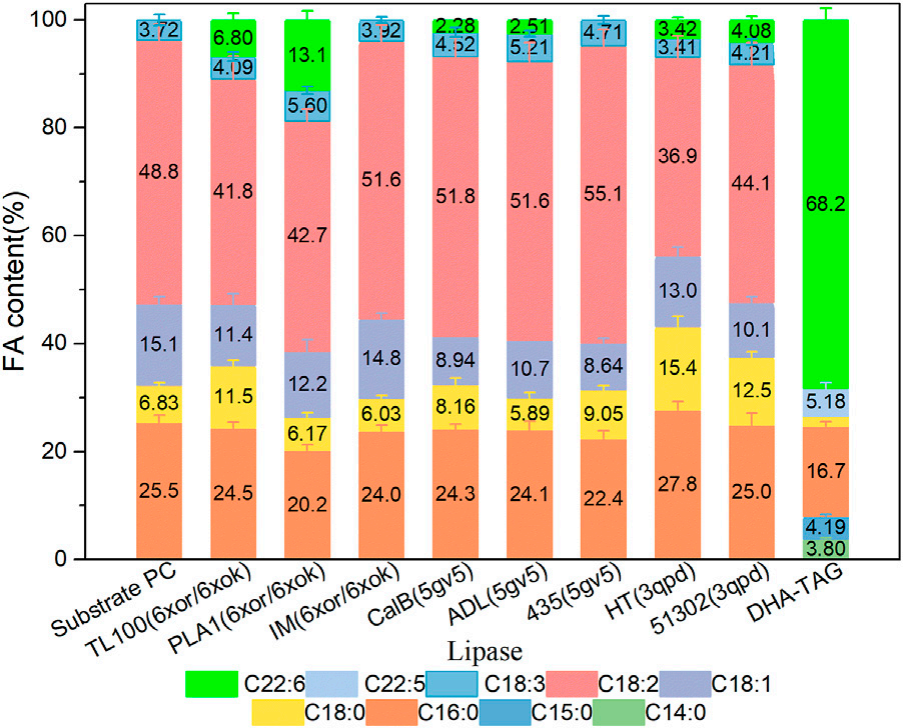
Profiles of FA in the product PC for different lipase-mediated transesterifications; Reaction conditions: 20 mg PC, 1.0 g algae oil, lipase (10 µL for liquid lipases and 20 mg for immobilized lipases), 45°C, 500 rpm, 1 h.

**FIGURE 4:**
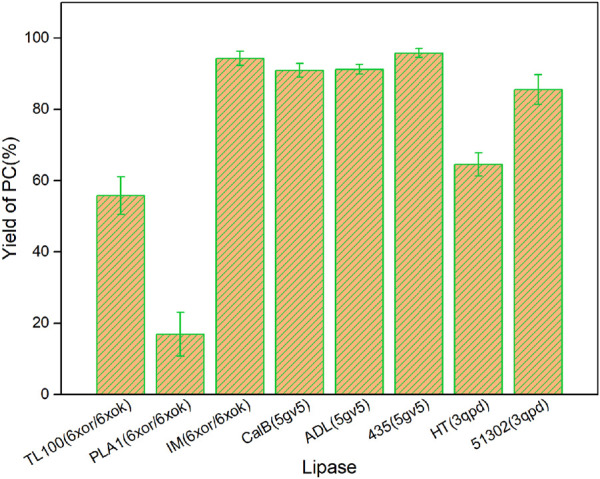
Yield of the product PC for different lipase-mediated transesterifications; Reaction conditions: 20 mg PC, 1.0 g algae oil, lipase (10 µL for liquid lipases and 20 mg for immobilized lipases), 45°C, 500 rpm, 1 h.

The catalytic performances of the eight commercial lipases containing six liquid lipases and two immobilized lipases were further evaluated by the transesterification of PLs with edible DHA-TAG from algae oil in a solvent-free system. Lipase could catalyze hydrolysis, esterification, and transesterification simultaneously. The fatty acid (FA) content and yield of PC in the product were used as parameters to measure the enzyme performance. The catalytic performances of different lipases are shown in Figures 3, 4. Lipases from *thermomyces lanuginosus* (TL100 and PLA1) exhibited higher DHA incorporation compared with the others. The highest DHA incorporation of 13.1% and the lowest PC yield of 16.9% were obtained for the PLA1-mediated transesterification. This could be explained by the molecular docking results. As shown in Figure 1, 6xok had the best affinity (−8.5 kcal/mol) for DHA compared with C18: 2 and C16: 0. Moreover, among all the selected lipases, lipase from *thermomyces lanuginosus* (PDB ID: 6xok) had the best affinity (−7.3 kcal/mol) with DUPC. The FA content in the product had a positive relation with the selectivity of the lipase for FA. The product PC catalyzed by lipases from *Moesziomyces antarcticus* (CALB and ADL) had a higher C18: 2 but lower DHA content, whereas lipases from *Aspergillus oryzae* had a lower DHA but higher C18: 0 content.

Herein, two kinds of immobilized lipase-mediated processes (IM and 435) without additional water were studied. They were almost inactive due to the little DHA content (less than 1%). A decrease in C16: 0 and an increase in C18: 2 contents were observed. It indicated that only a slight hydrolysis reaction may have occurred at the sn-1 position, which may attribute to the inherent moisture of the immobilized lipases. For the substrate PC, saturated fatty acid C16: 0 is mainly occupied the sn-1 position and unsaturated fatty acid C18: 2 is occupied the sn-2 position (Chojnacka et al., 2017). As a result, PLA1 was selected for the subsequent experiments and simulations due to the highest DHA incorporation.

### 3.3 Effect of water content on the PLA1-mediated transesterification

**FIGURE 5:**
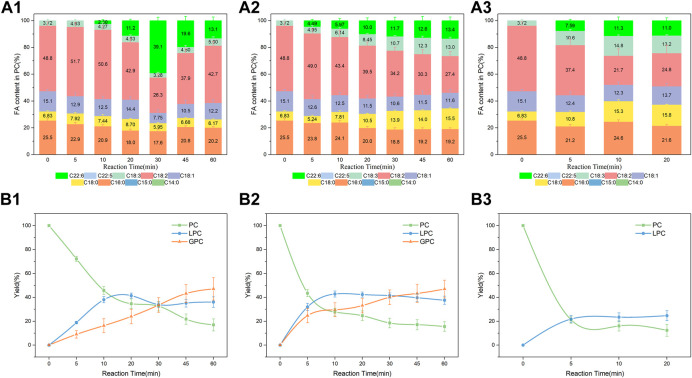
Effect of water content on the PLA1-mediated transesterification; Profiles of FA in the product PC containing **(A1)** 0.7 wt % water in the mixture; **(A2)** 1.2 wt % water in the mixture; **(A3)** 1.7 wt % water in the mixture; Yield of the product PC and LPC containing **(B1)** 0.7 wt % water in the mixture; **(B2)** 1.2 wt % water in the mixture; **(B3)** 1.7 wt % water in the mixture; Reaction conditions: 20 mg PC, 1.0 g algae oil, 10 µL PLA1, 45°C, 500 rpm. Notes: water herein refers to phosphate buffer solution (PBS, 0.1 M, pH 7.5).

Comparing the free and immobilized lipases we found that the presence of water was essential in this system, which was following the previous studies (Vikbjerg et al., 2005; Engel et al., 2016). The effect of micro-water content in PLA1-mediated transesterification of PC and DHA-TAG was further investigated. Results were shown in Figure 5. As shown in Figure 5A1, the incorporation of DHA increased dramatically with the reaction time before 30 min whereas decreased after 30 min at a water content of 0.7 wt%. The maximal incorporation of DHA (39.1%) was obtained at 30 min. The decrease after 30 min was due to the hydrolysis of the product DHA-PC, thereby producing LPC and GPC. The FA contents in LPC were also measured and the results were shown in Supplementary Figure S1. The lower C16: 0 content but higher C18: 2 content in the product LPC confirmed the selective hydrolysis of PLA1 occurred at sn-1 of PC (Vikbjerg et al., 2005; Engel et al., 2016). Moreover, the incorporation of DHA on LPC could be explained by the acyl migration phenomenon (Liu et al., 2017; Wang et al., 2020; Zhang et al., 2022). After the migration of the acyl group from the sn-2 position to the sn-1 position, the transesterification was proceeded catalyzing by PLA1 to obtain the sn-1 DHA-LPC. As shown in Figure 5B1, yields variations of PC, LPC and GPC with the reaction time conformed to the regularity of consecutive reaction. A sharp decrease of PC and a significant increase of LPC were observed at the initial stages due to the extensive hydrolysis reaction occurred.

When an additional 0.5 wt% water was added (total water content increased to 1.2 wt%), 80% of the PC had been hydrolyzed within 30 min and the incorporation of DHA in the product PC was only 11.7%. Only about 20% of the product PC was detected at 5 min when the total water content of 1.7 wt%, the rest of the PC was partly hydrolyzed to produce LPC and GPC, and partly precipitated from the mixture. PC is an amphiphilic substance. The certain amount of water attracted PC by hydrophilicity from the hydrophobic algal oil phase, as a result, partly PC would precipitate from the mixture before enzymatic reactions.

To sum up, transesterification and hydrolysis of PC and LPC occurred simultaneously within the reaction processing. A micro-water favored incorporating DHA into PC and LPC, whereas an extra addition of water for the free PLA1-mediated reaction had a negative effect on it.

### 3.4 Effect of temperature on the PLA1-mediated transesterification

**FIGURE 6:**
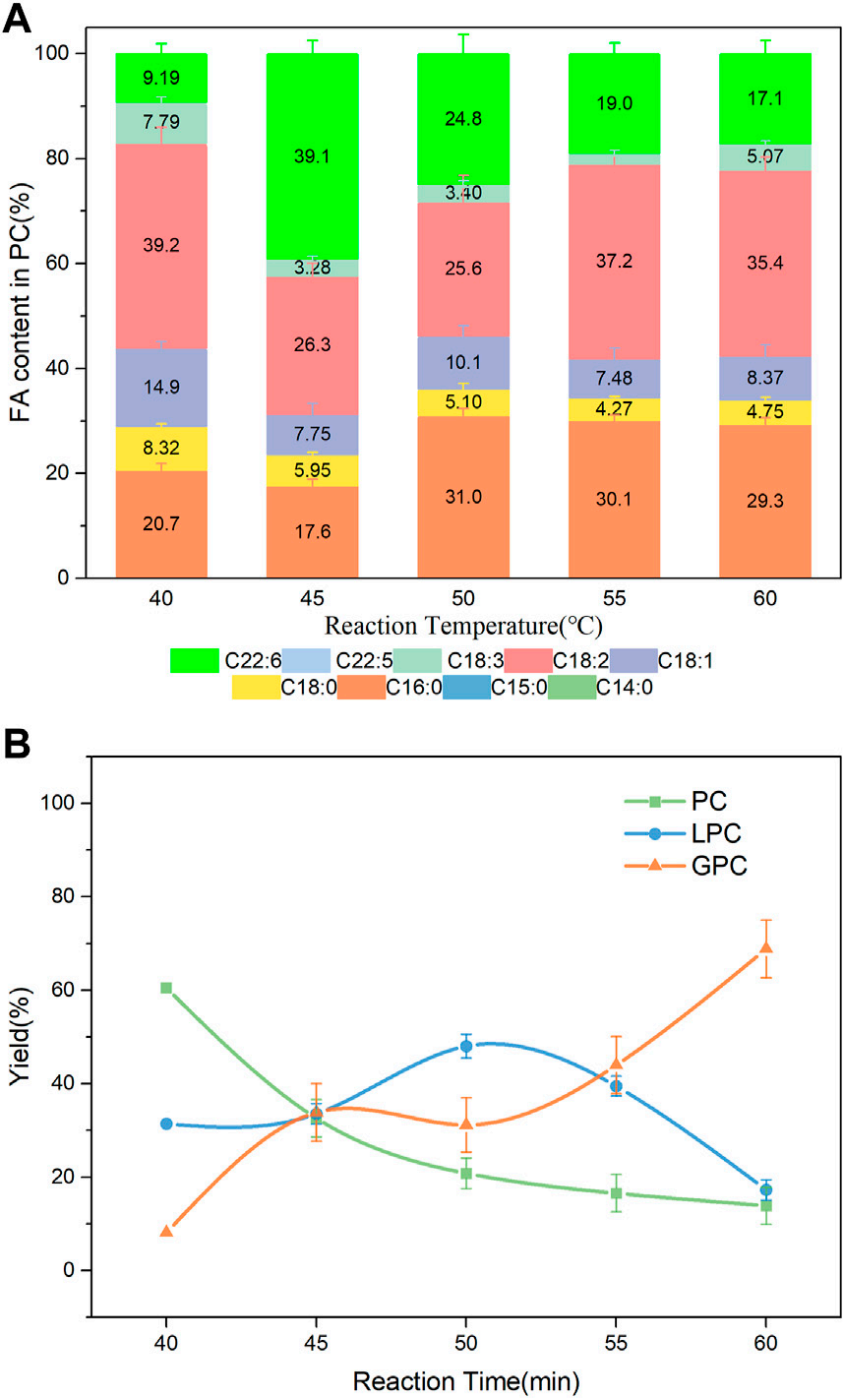
**(A)** Profiles of FA in the product PC; **(B)** Yield of the product PLs at different temperature; Reaction conditions: 20 mg PC, 1.0 g algae oil, 10 µL PLA1, 500 rpm, 30 min.

Temperature is crucial to the enzymatic transesterification. The solubility of PC in DHA-TAG increased and the viscosity of the mixture decreased with a higher reaction temperature. As shown in Figure 6; Supplementary Figure S2, compared with 40°C, higher incorporations of DHA in the product whereas lower total yields of the product PC and LPC were obtained. The DHA content was increased from 9.19% at 40°C to 39.1% at 45°C, and a slower decreased to 24.8% at 50°C. Reaction temperature is positively correlated with the hydrolysis rate. Excessively high temperature favored hydrolysis of PC than transesterification, which result in a reduce in DHA incorporation after 50°C. Moreover, it was observed that C16:0 content increased over 50°C maybe because PLA1 preferred C16: 0 at higher temperature.

### 3.5 PLA1-mediated transesterification of PC with different DHA donors

**FIGURE 7:**
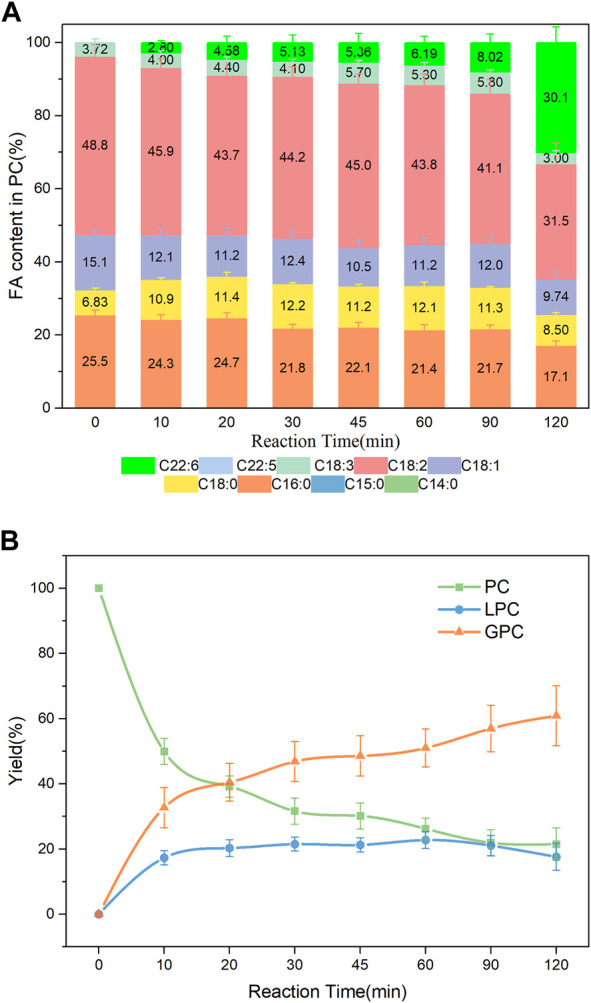
**(A)** Profiles of FA in the product PC; **(B)** Yield of the product PLs in PLA1-mediated transesterification of PC with DHA-ME; Reaction conditions: 20 mg PC, 1.0 g DHA-ME derivatized from algae oil, 10 µL PLA1, 45°C, 500 rpm.

The effects of three different DHA donors (DHA-TAG, DHA-ME, and DHA) on DHA incorporations were compared. DHA had better solubility for PLs but bad low-temperature fluidity. PC was barely hydrolyzed, but also DHA was barely incorporated (data were not shown). The possible reason is that the fatty acid form of DHA inhibited the activity of PLA1, resulting in poor hydrolysis and transesterification activity. Compared with DHA-TAG, DHA-ME with smaller steric hindrance had a good mutual solubility for PC and LPC. As shown in Figure 7, at the initial stages of reaction, the incorporation of DHA in PC increased slowly. But it is increased dramatically after 90 min, and a maximum DHA incorporation of 30.1% was obtained at 120 min, while lower yields (<20%) of both DHA-PC and DHA-LPC were produced. DHA-TAG had a higher steric hindrance and a bad solubility for PLs. However, PLA1 showed an excellent activity in both hydrolysis and transesterification, as shown in Figures 5A1, B1 and Supplementary Figure S1. On the one hand, PC was forced to dissolved in DHA-TAG by ultrasound. The amphipathic PC gathered on the oil-water interface when the free PLA1 (had a certain amount of water) was added and stirred evenly, making a higher PC concentration around the lipases and facilitating the contact between the enzyme and the substrates. On the other hand, PLA1 (PDB ID:6xok) showed an excellent performance with the glycerides of DHA as well as the DHA-ME (shown in Supplementary Table S3; Supplementary Figure S5). Although the glycerides of DHA had the large steric hindrance, their extreme hydrophobicity made it easier to enter the active pocket and interact with the active center of the lipase.

### 3.6 PLA1-mediated transesterification of different PLs with edible DHA-TAG

**FIGURE 8:**
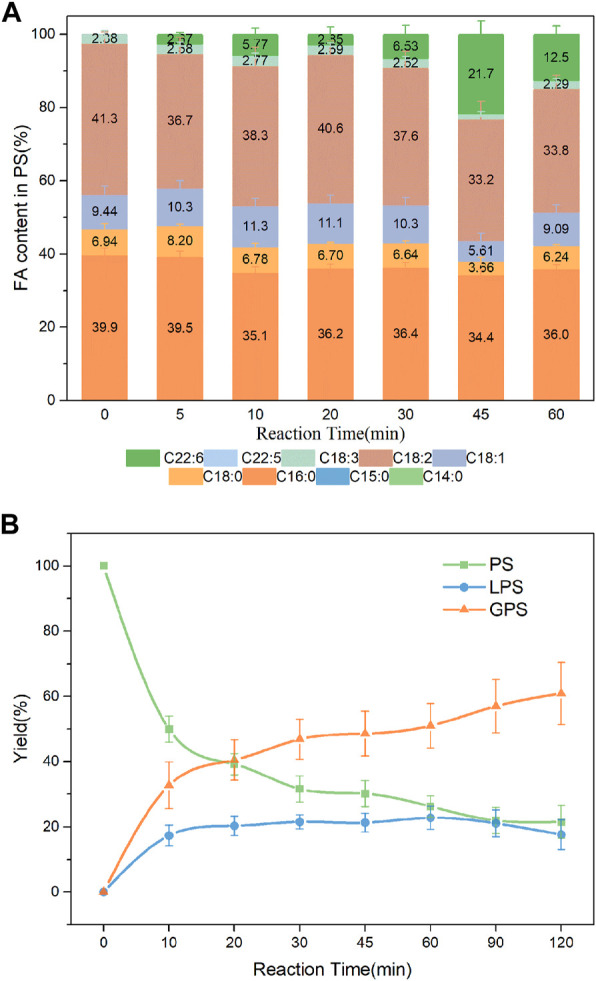
**(A)** Profiles of FA in the product PS; **(B)** Yield of the product in PLA1-mediated transesterification of PS with DHA-TAG; Reaction conditions: 20 mg PS, 1.0 g algae oil, 10 µL PLA1, 45°C, 500 rpm.

**FIGURE 9:**
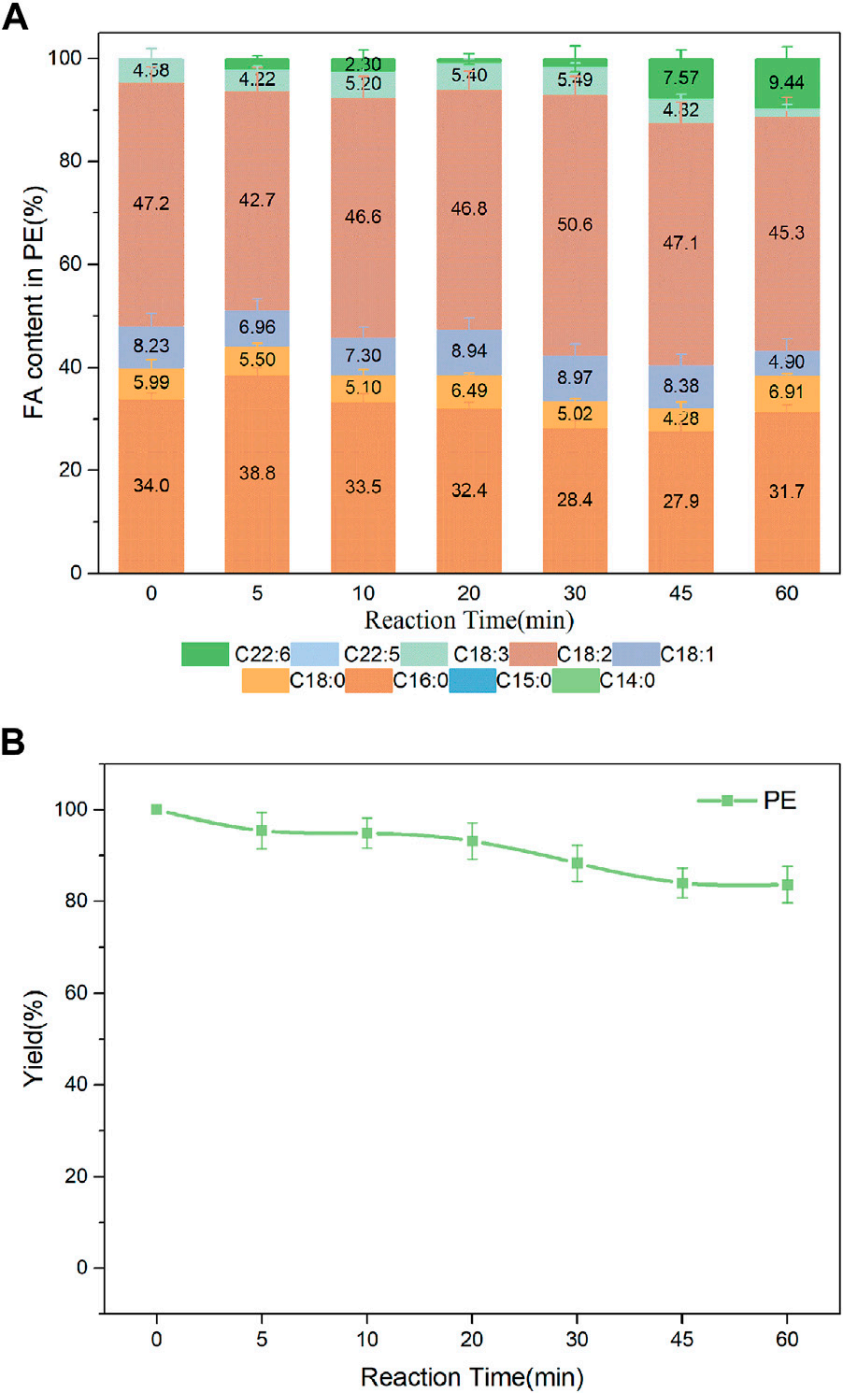
**(A)** Profiles of FA in the product PE; **(B)** Yield of PE in PLA1-mediated transesterification of PE with DHA-TAG; Reaction conditions: 20 mg PE, 1.0 g algae oil, 10 µL PLA1, 45°C, 500 rpm.

Studies suggested that DHA-PLs represented a potential novel therapeutic candidate for the treatment of neurodegenerative diseases such as AD, and that the polar group of the attached phospholipid was important to its bioactivity (Wen et al., 2016; Zhao et al., 2020). DHA enriched-PS/LPS and DHA enriched-PE were synthesized in the present work. As shown in Figure 8A, DHA incorporation in the product PS had an increase from 5 min to 45 min, whereas a slight decrease after that. The value reached a maximum of 21.7% at 45 min. Fortunately, higher incorporation of DHA in the product LPS was obtained shown in Supplementary Figure S4. Both the DHA incorporation and LPC yield reached a maximum of 24.8% and 34.8%, respectively at 30 min. It was observed from Figure 9 that when the substrates were PE and DHA-TAG, both hydrolysis and transesterification activities were insignificant.

### 3.7 Diffusions of substrates on PLA1 in the in solvent-free system

To further understand the mechanism of PLA1-mediated transesterification of PC and DHA-TAG in solvent-free system, two micro-units were re-constructed by MD under the periodic boundary condition to simulate the whole reaction system, containing micro-water and no water systems.

As shown in Supplementary Figures S6, S7, RMSD (Root Mean Squared Error) of distances between PLA1 and different substrates were calculated to quantitatively analyze the whole diffusion and binding processes in the solvent-free system. Until now, no PC or DHA-TAG were successfully co-crystalized with PLA1. Considering the huge steric resistance of the ligands and the difficulty of the free diffusion, the cutoff value of the crucial distance between the active pocket of PLA1 and its ligands was set as 15 Å to evaluate whether these ligands entered the active pocket in MD simulations.

Compared with the two systems, there is no significant difference in fluctuation in the RMSD of PC and DHA-TAG. Furthermore, there was always several DHA-TAG molecules entered the active pocket (RMSD <15 Å) and formed a stable PLA1—DHA-TAG complex during all the four times of MD. However, for the MRSD of PC, systems of micro-water and no water showed great differences. More PC molecules entered the active pocket when micro-water was used. Thus, the micro water was advantageous for the forming of PLA1—PC complex.

**FIGURE 10:**
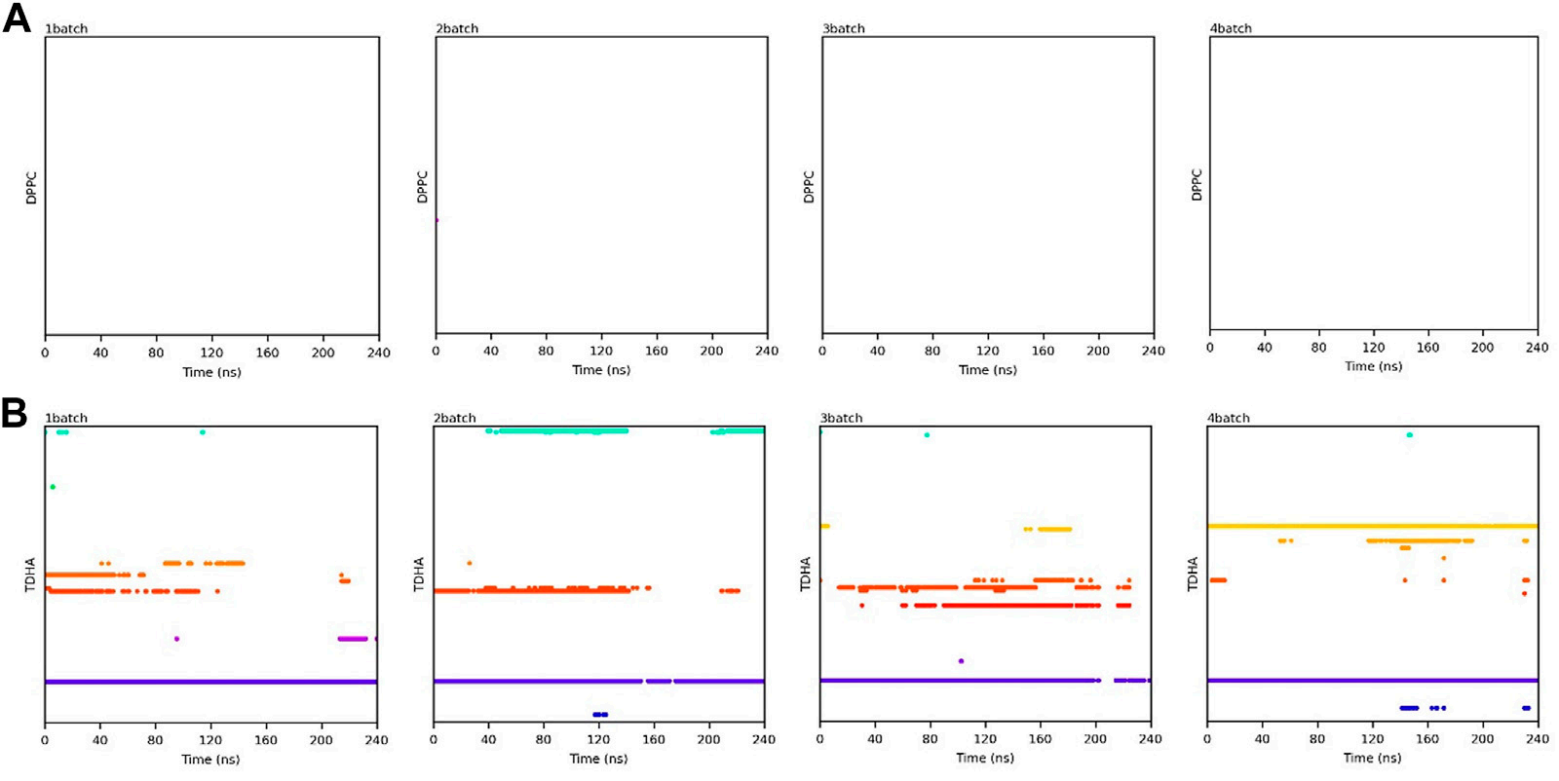
Binding time of the substrates **(A)** PC; **(B)** DHA-TAG in the active center of PLA1 in solvent-free system without water. Each color represents one substrate molecule. The length of the line with each color reflected the stability of the relevant complex; DPPC stands for PC and TDHA stands for DHA-TAG in the figure label.

**FIGURE 11:**
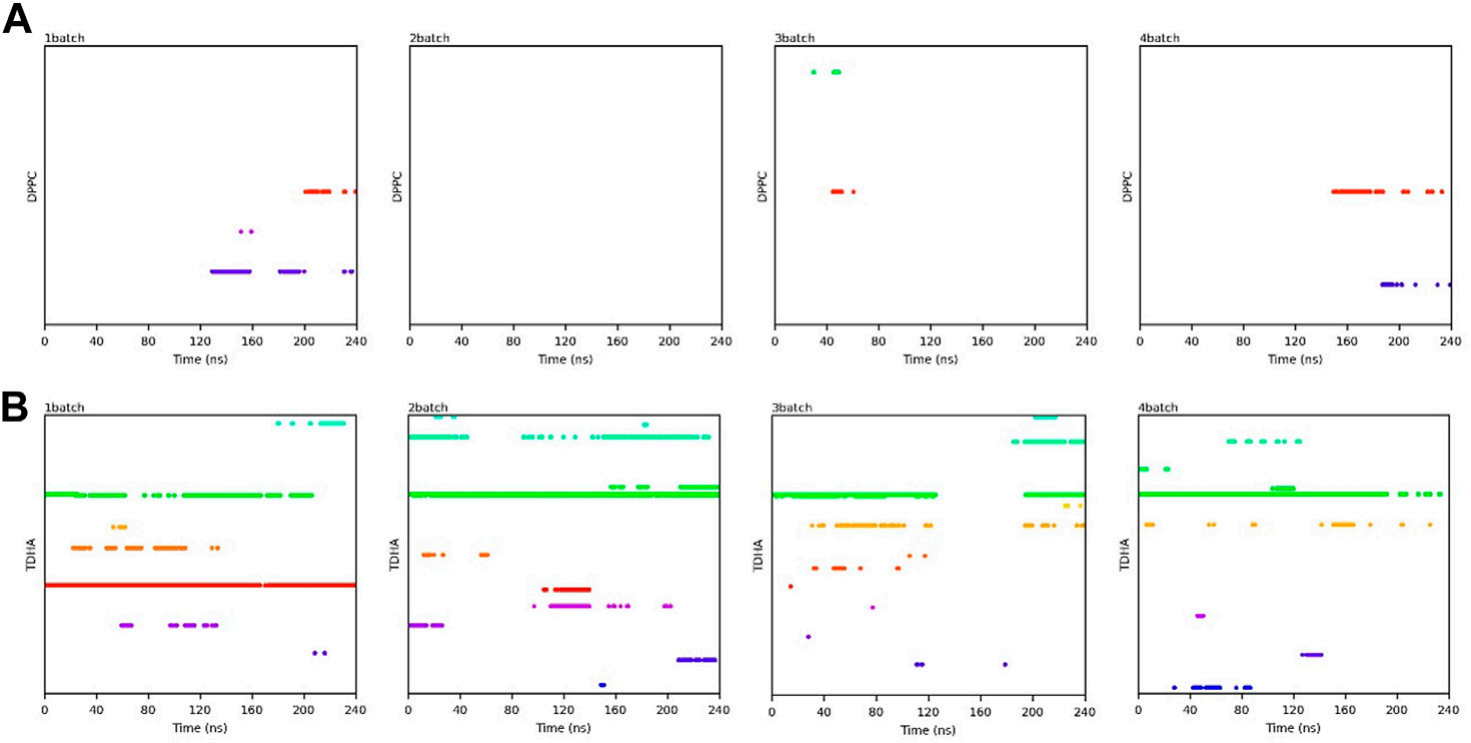
Binding time of the substrates **(A)** PC; **(B)** DHA-TAG in the active center of PLA1 in solvent-free system with micro-water. Each color represents one substrate molecule. The length of the line with each color reflected the stability of the relevant complex; DPPC stands for PC and TDHA stands for DHA-TAG in the figure label.

Transesterification was proceeded when PC and DHA-TAG were simultaneously bound in the active pocket of PLA1. The binding time profiles of each substrate molecule were systematically analyzed during each MD process (shown in Figures 10, 11). The overlapping binding time coordinates between the two substrates could reflect the formation of the PLA1—PC—DHA-TAG complex. Although PLA1—DHA-TAG complexes could be formed in the anhydrous system (Figure 10B), some of them could not further interact with PC molecules and form the PLA1—PC—DHA-TAG complex (Figure 10A). However, at least two PC molecules (except 2batch) could be overlapping with that of DHA-TAG and, thus, more PLA1—PC—DHA-TAG complexes could form in the micro-water system (Figures 11A, B), which should be beneficial for the PLA1-mediated transesterification of PC and DHA-TAG in solvent-free system. It could be concluded that the addition of trace amounts of water facilitated the entry of PC molecule into the active pocket, while the effect on DHA-TAG was not significant. Similar findings can be observed in Table 3. Five times higher of the average total numbers of PC entering the active pocket in the micro-water system than that of anhydrous system. The binding time in the micro-water system was much higher than that of in the anhydrous system.

**TABLE 3:** The number of the substrates entering the active center and binding time.

	Substrates	System	1batch	2batch	3batch	4batch	Average
Total number of the substrates entering the active center	PC	Anhydrous	0	2	0	0	0.5
		Micro-water	4	0	3	3	2.5
	DHA-TAG	Anhydrous	10	8	11	10	9.75
		Micro-water	9	13	13	9	11
Total binding time [ns]	PC	Anhydrous	0	0.01	0	0	0.0025
		Micro-water	37.64	0	3.34	22.45	15.86
	DHA-TAG	Anhydrous	304.21	408.44	338.03	324.46	343.785
		Micro-water	364.71	546.06	255.12	216.03	345.48

**FIGURE 12:**
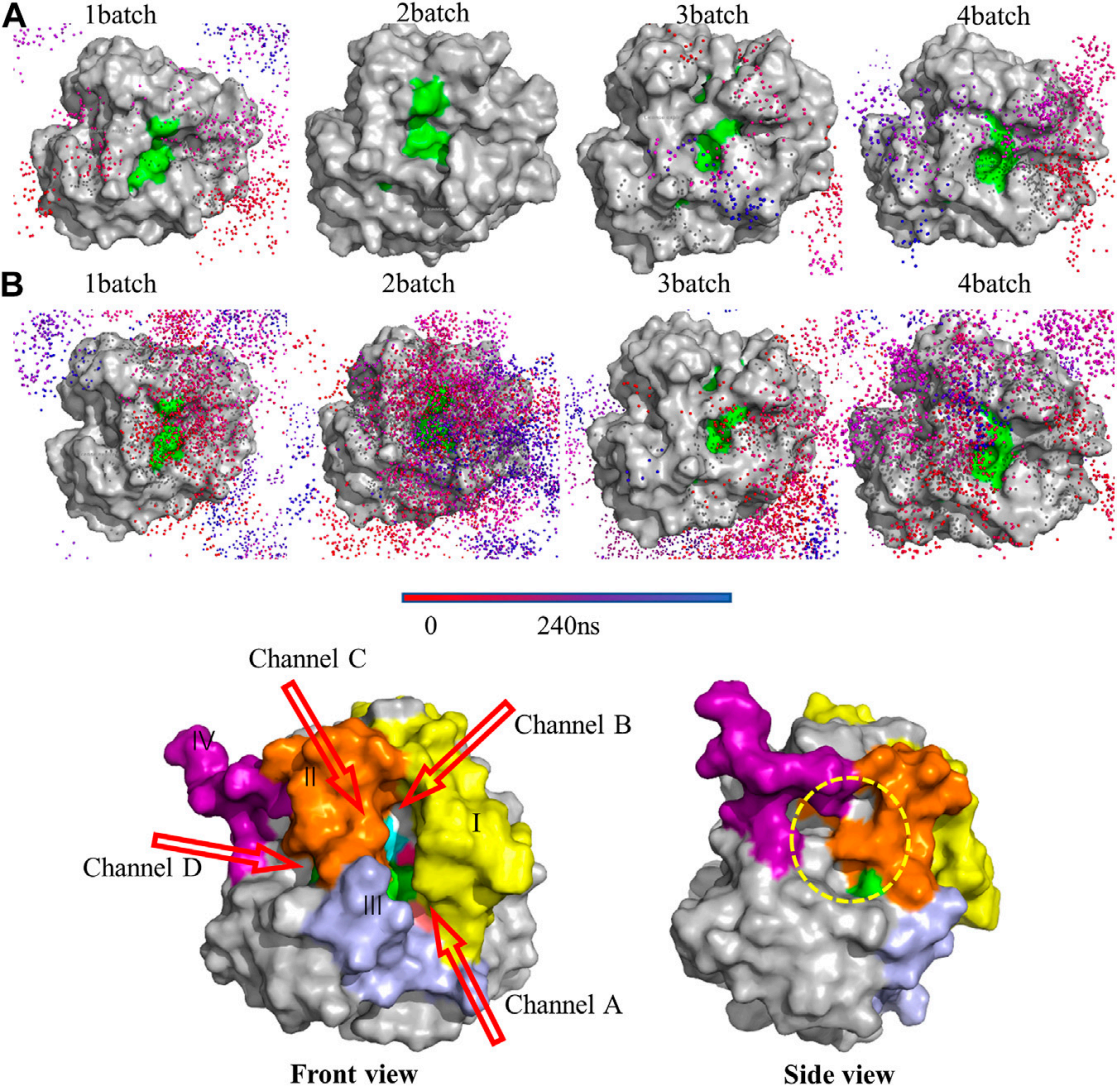
Trajectories of the substrates **(A)** PC; **(B)** DHA-TAG in solvent-free system with micro-water under 4 MD simulation; Only trajectories of the substrates which could enter the active pocket were shown. Other molecules were hidden for the easier visualization. The catalytic triad residues (S146-D201-H258) were represented as green color to guide the location of the active pocket; **(C)** The diffusional channels of PLA1. Four main regions with obvious changes were shown by different colors. The catalytic triad residues (S146-D201-H258) were represented as green to guide the active pocket’s location.

Trajectories of the substrates on the PLA1 surface were visualized to investigate the binding pathways and kinetic behaviors, as shown in Figure 12; Supplementary Figure S8. The aggregations and densities of PC and DHA-TAG could be used to reflect their affinities with PLA1. Apparently, PC showed lower affinity with PLA1 without water. In contrast, when micro-water was used, the obvious aggregations of PC molecular trajectories were observed. It was implied that micro-water facilitated the interaction between PC and PLA1.

### 3.8 Structure activity relationships of the PLA1

RMSD of each residue from the PLA1 backbone was calculated in all frames of MD simulations, as shown in Supplementary Figure S9. In general, the structure of proteins is more flexible in aqueous media. Because the systems in the present work were anhydrous and micro-water, the residues in this protein had a small variation. Residues at regions with relative flexibility (RMSD >0.025 Å) were focused.

High flexible residue regions were close to the active pocket of the PLA1, involving region Ⅰ (79-106, SFRGSRSLENWIGNLNFDLKEINDICSG in yellow), region Ⅱ (202-216, IVPRLPPREFGYSHS in orange), region Ⅲ (253-269, PDIPAHLWYFGLIGTCL in light purple), and region Ⅳ (238-244, IRGIDAT in magenta) as shown in Figure 12C. Combined with the diffusional trajectories of the substrates (Figure 12; Supplementary Figure S8), four main diffusional channels were confirmed. Channels of A, B, and C were converged above the front of active center and channel D was converged above the side of active center. The diffusional channel A was composed of regions Ⅰ and Ⅲ, the diffusional channel B was in region Ⅰ, channel C was in regions Ⅱ, and channel D located at the side of the active pocket (the yellow dotted circle).

According to the diffusional trajectories (shown in Figure 12; Supplementary Figure S8), the most important pathway was channel A and channel C due to its widest size and lowest hindrance. Channel B did not change much during the whole transesterification. Although there were several substrate molecules entering the active center through channel D, the spatial changes of channel D were frequent and large due to the large number of loop structures near channel D. As a result, channel D was easily blocked. Thus, the regional changes among channel A and C would directly affect the activity and selectivity of PLA1.

**FIGURE 13:**
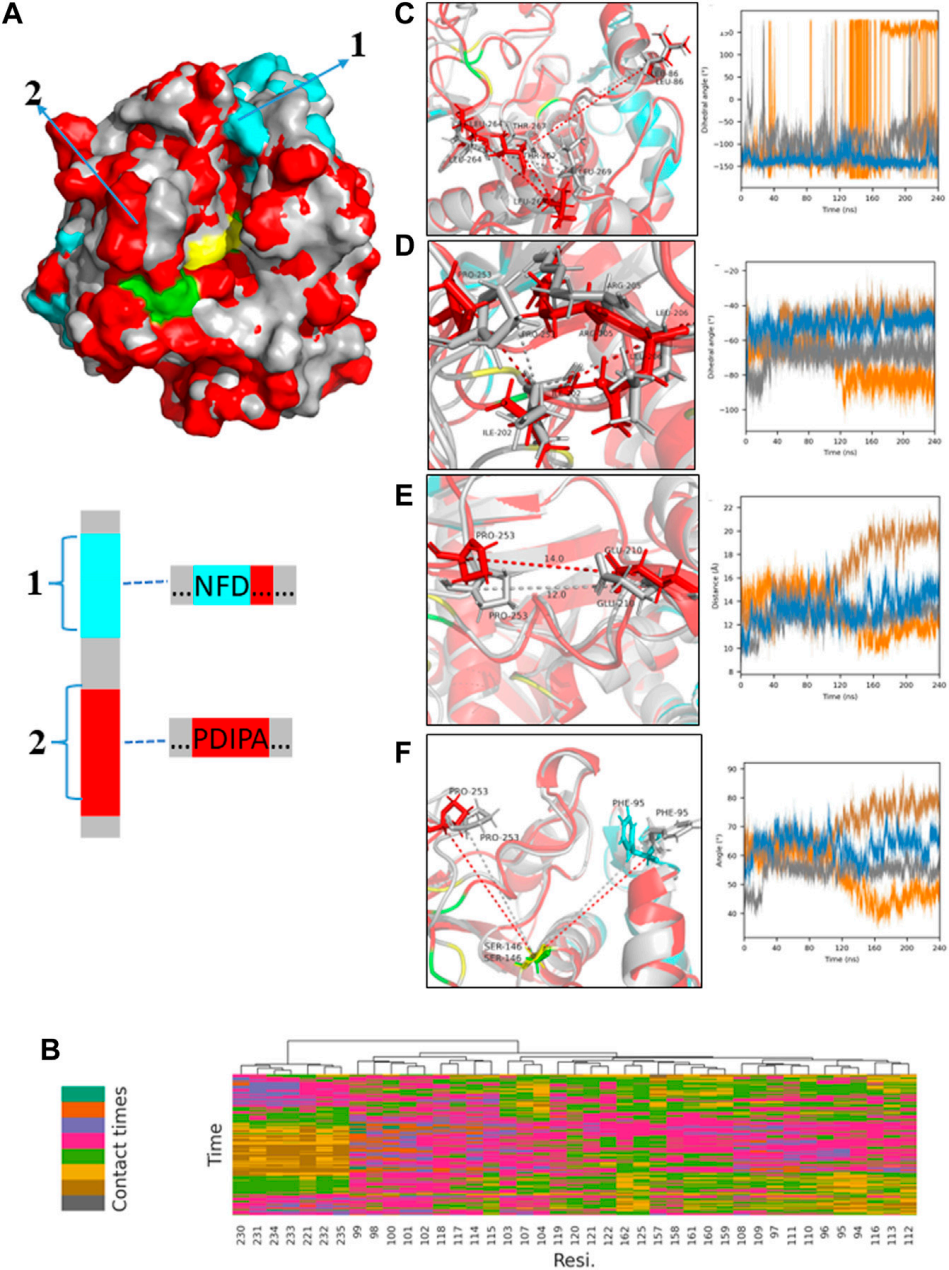
MD results for the micro-water system; **(A)** Comparison of the two protein molecular conformations of the original PLA1 (gray) and the PLA1 in the process of diffusion (red and cyan); the marked regions (1,2; amino acid sequences have been given) show the significant differences between the two PLA1 conformations and the cyan regions represent the better affinities with PC and DHA-TAG according to the results of **(B)**; **(B)** Statistics of average interaction frequencies of each residue with substrate molecule DHA-TAG and PC; The cutoff value was used to highlight the residues which have stronger affinities with substrates, which were set as 70. If the total interaction frequency of a residue with substrate during the whole simulation time was less than the cutoff value, this residue was hidden for the easier visualization. The interaction distance was set as 8 Å due to a larger steric hindrance; **(C)** Time evolution of the dihedral angle among L264, L269, T267, and L86 and its visualization result; **(D)** Time evolution of the dihedral angle among P253, I202, L206, and R205 and its visualization result; **(E)** Time evolution of the distance between P253 and E210 and its visualization result; **(F)** Time evolution of the angle among P253, S146 and F95 and its visualization result.

Figure 13; Supplementary Figure S10A showed the conformational change of PLA1 during diffusion. The strong affinity residues (colored cyan) mainly located in channel C, implying that the change in channel C was mainly caused by the interaction of the substrate with PLA1 during diffusion. In contrast, changes in the spatial dimensions of the key channel A were less affected by the substrate. As shown in Figure 13C; Supplementary Figure S10C, changes in the dihedral angle of L264, L269, T267, and L86 were mainly determined by the movement of L264 and L86. This value directly reflected the dimensions of channel A. The value either remains at 0° or ±180° in the anhydrous system, whereas it maintained from −150° to −50° with micro-water existing. It was implied that channel A in the micro-water system was always maintained in a wider dimension. It is beneficial to the substrates DHA-TAG and PC diffuse into the active pockets. The dihedral angle among P253, I202, L206, and R205 reflected the open and closed state of channel C. It was observed from Figure 13D; Supplementary Figure S10D that channel C in the micro-water system was more suitable for diffusing into the active pocket, while it was potentially narrower in the anhydrous system. Distance between P253 and E210 shown in Figure 13E exhibited a bigger value varied from 10 Å to 16 Å, while under the anhydrous situation the variation of its value was only 2 Å (Supplementary Figure S10E). Bigger distance meant lager dimensions of channel C. Furthermore, the angle among P253, S146 and F95 was also an important parameter for the size of the channel C shown in Figure 13F and Supplementary Figure S10F. The variation of the angle was mainly caused by the movement of P253 and F95. This value in the micro-water system varied from 40° to 70°, while the value in the anhydrous system always remained in a lower range (all values were fluctuated around 40° except for one batch). In addition, in Figure 13A, the green-colored H258 was closer to the PLA1 surface than its initial position (yellow-colored), while S146 and D201 were essentially unshifted, suggesting a larger range of the active pocket composed of S146-D201-H258 in the micro-water system.

## 4 Conclusion

The structure PLs of DHA-PC, DHA-PS and DHA-PE were prepared from PLA1-mediated transesterification of PLs with edible DHA-TAG from algae oil in a solvent-free system. We virtually screened 153 protein structures of triacylglycerol lipase. PLA1 (PDB:6xok) was proven to be the best candidate due to a higher DHA incorporation. The reaction temperature and water content were optimized. A maximum incorporation of DHA in PC was reached 39.1% at 45°C and 0.7% water content (without additional water addition). The different DHA donors including forms of fatty acid, methyl ester, and triglycerides were compared. MD results had explained the higher transesterification and hydrolysis activity of PLA1 in the micro-water system at the molecular level.

## Data Availability

The original contribution presented in the study are included in the article/Supplementary Material, further inquiries can be directed to the corresponding authors.
