# Supplementary material for: Green biosynthesis of rare DHA-phospholipids by lipase-catalyzed transesterification with edible algal oil in solvent-free system and catalytic mechanism study
Source: Front Bioeng Biotechnol. 2023 Mar 31;11:1158348. doi: 10.3389/fbioe.2023.1158348 (PMC10102545; doi:10.3389/fbioe.2023.1158348)
Supplement: Supplementary file 1 [file DataSheet1.docx]

Supplementary Information

Green biosynthesis of rare DHA-phospholipids by lipase-catalyzed transesterification with edible algal oil in solvent-free system and catalytic mechanism study

Tiantian Zhang^a,#^, Binglin Li^a#^ , Zhulin Wang^a^, Dan Hu^a^, Xiaoli Zhang^a*^, Binxia Zhao^b^, Jiao Wang^c,d^*

a College of Food Science and Engineering, Northwest University, Xi’an 710069, China

b College of Chemical Engineering, Northwest University, Xi'an 710069, China e Logistics Group, Northwest University, Xi'an 710069, China

c Biochemistry Center (BZH), Heidelberg University, Heidelberg 69120, Germany

d BioQuant, Heidelberg University, Heidelberg 69120, Germany

# T. Z. and B.L. contributed equally to this work.

* Corresponding authors:

Xiaoli Zhang Email: xlzhang@nwu.edu.cn, ORC-ID: 0000-0003-3702-3700

Jiao Wang Email: jiao.wang@bioquant.uni-heidelberg.de

Table S 1 Original affinity (kcal/mol) data of the molecular docking

| Ligand  PDB ID | DHA-TAG | 2,3-DAG | 2-MAG | DHA | C16:0 | C18:2 | DPPC | DUPC |
| --- | --- | --- | --- | --- | --- | --- | --- | --- |
| 1i6w | -3.8 | -3.4 | -3.4 | -4.7 | -3.4 | -3.1 | -3.7 | -3.8 |
| 1r4z | -3.4 | -4.2 | -4.2 | -4.3 | -3.4 | -4.3 | -4.2 | -3.8 |
| 1r50 | -3.3 | -4.1 | -3.8 | -3.9 | -3.8 | -3.5 | -3.5 | -3.9 |
| 1t2n | -4 | -3.3 | -3.8 | -3.4 | -3.8 | -4 | -3 | -3.3 |
| 1t4m | -2.6 | -3 | -3.9 | -3.3 | -3.1 | -3.4 | -3.4 | -3.4 |
| 2qxt | -3.9 | -3.7 | -4.7 | -3.4 | -3.1 | -4.3 | -3 | -2.9 |
| 2qxu | -3.3 | -3.4 | -3.3 | -3.5 | -3.4 | -3.7 | -4.2 | -3.6 |
| 3d2a | -3.2 | -3.8 | -4.4 | -4.2 | -3.7 | -3.3 | -3.4 | -4 |
| 3d2b | -2.6 | -4.1 | -3.9 | -3.7 | -3.6 | -4.2 | -3.2 | -2.9 |
| 3d2c | -3.5 | -3.3 | -3.9 | -3.6 | -3.1 | -3.3 | -3.1 | -3.3 |
| 3qmm | -2.4 | -3.3 | -4.2 | -4.9 | -3.9 | -3.6 | -3.5 | -3.7 |
| 3qzu | -3.2 | -3.3 | -4.2 | -4.8 | -3.6 | -4.2 | -3.7 | -3.2 |
| 5cri | -4.5 | -3.9 | -3.8 | -4 | -3.4 | -4.2 | -4 | -4.5 |
| 5ct4 | -4.4 | -3.6 | -4.7 | -3.7 | -4.1 | -4.2 | -4.4 | -4.1 |
| 1hqd | -6.6 | -6.4 | -5.8 | -7.1 | -4.7 | -6.2 | -4.9 | -5.8 |
| 1oil | -6.6 | -5.9 | -5.4 | -6.6 | -5.1 | -6.2 | -5.4 | -5.4 |
| 1ys1 | -7.1 | -7.6 | -6.4 | -7 | -5.6 | -6.3 | -6 | -5.7 |
| 1ys2 | -7.4 | -6.8 | -6.3 | -6.4 | -5.3 | -6.2 | -5.8 | -6.3 |
| 2lip | -6.4 | -5.4 | -6.7 | -6.3 | -4.5 | -4.6 | -5.1 | -5.6 |
| 2nw6 | -7 | -6.9 | -6.5 | -6.3 | -5.8 | -6.4 | -5.9 | -6.1 |
| 4lip | -7.4 | -6.4 | -6.2 | -6.5 | -6.1 | -6.7 | -6.1 | -6.1 |
| 5lip | -7 | -7.1 | -6.7 | -6.4 | -5.1 | -6.6 | -5.4 | -5.7 |
| 1crl | -5.4 | -4.9 | -5.8 | -5.1 | -4.5 | -5 | -5.1 | -4.9 |
| 1lpm | -6 | -5.1 | -6.6 | -6.5 | -5.4 | -6 | -5.6 | -6.1 |
| 1lpn | -4.9 | -6.7 | -6.2 | -6.8 | -6.1 | -6.5 | -6.2 | -6.3 |
| 1lpo | -6.8 | -5.6 | -6.2 | -5.2 | -5.6 | -5.8 | -6.1 | -6.1 |
| 1lpp | -7.7 | -6.9 | -6.2 | -6.3 | -5.8 | -7 | -6.5 | -6.6 |
| 1lps | -5.3 | -5.9 | -6.1 | -5.7 | -5.3 | -6.3 | -5.8 | -5.7 |
| 3rar | -5.1 | -5.2 | -6.6 | -6.4 | -5.3 | -5.6 | -5.5 | -5.1 |
| 1f6w | -5.4 | -4.9 | -4.8 | -5 | -4.2 | -4.5 | -4.6 | -5.8 |
| 1jmy | -6.4 | -5 | -5.2 | -6.2 | -5.2 | -5.7 | -5.5 | -5.6 |
| 1lpa | -6 | -5.6 | -6.4 | -6.7 | -5.6 | -6.8 | -5.5 | -5.9 |
| 3w9u | 7.6 | -4.4 | -7.3 | -7.4 | -5.5 | -6 | -3.3 | -1.5 |
| 4gw3 | -4.8 | -7.4 | -7.3 | -7.2 | -5.3 | -6.1 | -5.6 | -6.2 |
| 4gxn | 5.8 | -6.7 | -7.3 | -6.5 | -4.7 | -6 | -6.3 | -6.1 |
| 6jd9 | -1.3 | -6.9 | -7.3 | -7 | -5.4 | -6.6 | -5.6 | -4.5 |
| 2zvd | -6.8 | -8.3 | -6.5 | -6.2 | -5.4 | -6.2 | -6 | -6.7 |
| 3a6z | -7.4 | -6.6 | -7.3 | -7.3 | -5.6 | -6.4 | -5.4 | -6.6 |
| 3a70 | -7.4 | -7.4 | -6.6 | -6.7 | -5.2 | -5.6 | -6 | -5.9 |
| 6zl7 | -7.4 | -6.5 | -6.6 | -6.2 | -4.8 | -5.1 | -5.6 | -6.4 |
| 6qin | -6 | -5.9 | -5.9 | -5.8 | -4.5 | -5.9 | -5.7 | -5.6 |
| 6qla | -6 | -6.4 | -5.7 | -5.9 | -4.7 | -5.3 | -5.6 | -6 |
| 1akn | -6 | -5.3 | -5.1 | -5.8 | -4 | -4.6 | -4 | -4.5 |
| 1aql | -6.6 | -5.9 | -7.5 | -7.8 | -5.3 | -7.1 | -6 | -6.5 |
| 1eth | -5.4 | -5.8 | -7.2 | -7.3 | -5.7 | -6.2 | -5.2 | -5.3 |
| 1ex9 | -6.6 | -7 | -5.5 | -5.7 | -4.3 | -5.5 | -5.8 | -5.1 |
| 1gpl | -5 | -4.8 | -6.8 | -6.4 | -5.5 | -6.1 | -5.6 | -5.6 |
| 1k8q | -5.4 | -8 | -7.6 | -7 | -5.3 | -6.4 | -4.8 | -6.6 |
| 1llf | -7.3 | -8.7 | -7.4 | -7.1 | -6.7 | -6.3 | -6.5 | -7.1 |
| 2hih | -5.3 | -5.7 | -5.2 | -5.2 | -3.9 | -4.4 | -4.5 | -5.1 |
| 2qua | -7 | -7 | -6.6 | -6.3 | -5.4 | -5.6 | -5.8 | -6.7 |
| 2qub | -6.8 | -5.3 | -6 | -5.6 | -5.2 | -5.7 | -6.1 | -6.4 |
| 2w22 | -6.7 | -6.8 | -6.6 | -6.7 | -5.5 | -6.1 | -6 | -6 |
| 3g7n | -5.2 | -4.8 | -4.4 | -3.7 | -3.9 | -5 | -3.7 | -4.7 |
| 5ce5 | -7.3 | -6.7 | -7.3 | -7.4 | -6.4 | -6.9 | -5.7 | -6.8 |
| 5mal | -4.3 | -5.5 | -4.5 | -4.9 | -4 | -4.8 | -4.6 | -4.5 |
| 3gbs | -4.9 | -5.8 | -5 | -5.1 | -4.3 | -4.8 | -4.7 | -4.6 |
| 3qpd | -5.1 | -5.9 | -5.4 | -5.6 | -4.6 | -4.9 | -5 | -5 |
| 5gv5 | -5.1 | -5.5 | -6.4 | -6.4 | -5.8 | -5.2 | -4.9 | -5.1 |
| 4k5q | -3.7 | -4.6 | -4.7 | -5.8 | -3.7 | -4.8 | -4 | -4 |
| 4k6g | -4.1 | -4.5 | -4.9 | -5.7 | -3.9 | -5.1 | -4 | -4.3 |
| 4k6h | -4.8 | -4.9 | -5.1 | -5.6 | -4.5 | -5 | -4.2 | -4.3 |
| 4k6k | -4.3 | -5.1 | -4.7 | -5.5 | -4.5 | -4.9 | -4.2 | -4.1 |
| 6j1p | -5 | -5.6 | -5.4 | -5.8 | -4.7 | -5.4 | -4.8 | -4.5 |
| 6j1r | -4.4 | -4.6 | -5.1 | -5 | -4.5 | -4.7 | -4.5 | -4.5 |
| 6j1q | -4.8 | -4.9 | -5.2 | -5.6 | -5 | -5.8 | -4.9 | -5.1 |
| 6j1t | -4.9 | -5.1 | -5.3 | -5.8 | -4.3 | -5.2 | -4.5 | -4.5 |
| 6j1s | -5.1 | -5.3 | -4.8 | -4.9 | -5 | -4.6 | -5.6 | -4.9 |
| 6isq | -5.8 | -5.6 | -5.5 | -5.9 | -4.8 | -4.8 | -5.2 | -5.4 |
| 6isr | -4.9 | -4.2 | -4.7 | -5.2 | -4.2 | -4.8 | -5 | -4.1 |
| 3w9b | -5.8 | -5.5 | -5.8 | -5.8 | -5.2 | -5.6 | -5 | -4.4 |
| 4zv7 | -5.2 | -5.3 | -5.8 | -6 | -5.5 | -6.5 | -4.7 | -4.3 |
| 6tp8 | -5 | -5.7 | -5.8 | -6.2 | -5.7 | -6 | -4.5 | -4.9 |
| 3icv | -4.5 | -4.4 | -4.2 | -4.9 | -3.9 | -4.7 | -4 | -4.4 |
| 3icw | -4.4 | -5.2 | -4.3 | -4.8 | -4.1 | -4.8 | -4.5 | -4.6 |
| 1tcc | -4.8 | -5.5 | -5.8 | -5.7 | -5.2 | -5.8 | -4.6 | -4.7 |
| 1tcb | -4.9 | -4.8 | -6 | -6.4 | -5.2 | -5.5 | -3.9 | -5.2 |
| 1tca | -5 | -5.7 | -5.1 | -6.3 | -5.1 | -5.6 | -4.3 | -4.7 |
| 1lbt | -4.9 | -6.1 | -6 | -6.8 | -5.1 | -5.3 | -4.6 | -5.1 |
| 1lbs | -4.6 | -5.1 | -5.6 | -6.4 | -5.2 | -5.4 | -4.5 | -4.8 |
| 6xrv | -6.4 | -6.2 | -5.5 | -6.1 | -5.7 | -6 | -5.9 | -5.5 |
| 6xok | -6.6 | -8.3 | -7.3 | -8.5 | -6.6 | -7.4 | -6.8 | -7.3 |
| 6or3 | -5.7 | -5.9 | -7 | -6.4 | -5 | -5.8 | -5.4 | -6 |
| 5a71 | -6.4 | -6.4 | -7.2 | -5.9 | -5.6 | -6.1 | -5.2 | -6 |
| 5a6v | -4.6 | -4.6 | -5.6 | -6.2 | -4.9 | -5.4 | -4.5 | -4.5 |

Table S 2 Original distance (Å) data of the molecular docking

| Ligand  PDB ID | DHA-TAG | 2,3-DAG | 2-MAG | DHA | C16:0 | C18:2 | DPPC | DUPC |
| --- | --- | --- | --- | --- | --- | --- | --- | --- |
| 1i6w | 3.6 | 4.4 | 4 | 3.8 | 3.9 | 3.6 | 3.9 | 4.2 |
| 1r4z | 3.5 | 4.4 | 4.9 | 3.9 | 3.9 | 3.7 | 3.5 | 3.6 |
| 1r50 | 3.1 | 3.6 | 3.5 | 4 | 3.6 | 3.7 | 3.4 | 3.5 |
| 1t2n | 3.8 | 4.3 | 3.8 | 4.7 | 3.6 | 3.6 | 3.7 | 3.8 |
| 1t4m | 4.8 | 6.9 | 19.3 | 3.7 | 3.6 | 3.5 | 3.5 | 4.1 |
| 2qxt | 3.6 | 4.2 | 3.7 | 4 | 4.1 | 3.7 | 3.6 | 3.6 |
| 2qxu | 3.6 | 4.3 | 4.2 | 6.1 | 4.2 | 3.6 | 4.3 | 3.8 |
| 3d2a | 3.7 | 3.6 | 6.7 | 4.1 | 4.2 | 3.8 | 3.8 | 3.7 |
| 3d2b | 3.5 | 4 | 3.8 | 4.4 | 3.6 | 3.6 | 3.7 | 3.5 |
| 3d2c | 3.6 | 4 | 4.1 | 3.3 | 3.8 | 3.6 | 3.5 | 3.4 |
| 3qmm | 3.3 | 5.2 | 5.5 | 4.9 | 3.9 | 3.8 | 3.7 | 3.6 |
| 3qzu | 4.2 | 5 | 8.4 | 3.8 | 4.2 | 3.5 | 3.6 | 3.8 |
| 5cri | 2.9 | 2.9 | 3.6 | 3.3 | 3.5 | 3.3 | 3 | 3.5 |
| 5ct4 | 3.1 | 3.4 | 3.3 | 3.5 | 3.3 | 3.4 | 3.3 | 3.7 |
| 1hqd | 3.8 | 4.2 | 3.8 | 3.6 | 3.9 | 3.6 | 3.7 | 5.4 |
| 1oil | 3.5 | 3.6 | 4.3 | 3.5 | 4.2 | 3.7 | 4.1 | 4.8 |
| 1ys1 | 3.7 | 3.9 | 3.9 | 3.6 | 3.8 | 3.6 | 3.8 | 4.7 |
| 1ys2 | 3.7 | 3.4 | 3.8 | 3.9 | 3.8 | 3.5 | 3.3 | 3.8 |
| 2lip | 4 | 4.1 | 3.9 | 4 | 4 | 4.3 | 3.8 | 4.2 |
| 2nw6 | 3.7 | 3.7 | 3.5 | 3.7 | 3.7 | 3.8 | 3.8 | 4 |
| 4lip | 3.7 | 3.8 | 4.8 | 3.8 | 3.9 | 4.1 | 3.9 | 6 |
| 5lip | 3.9 | 3.8 | 4 | 3.9 | 3.8 | 4.2 | 3.8 | 6 |
| 1crl | 4 | 4.5 | 4.6 | 3.7 | 3.6 | 3.7 | 4.2 | 4 |
| 1lpm | 3.8 | 3.9 | 4.5 | 3.7 | 3.8 | 3.9 | 4.1 | 4.8 |
| 1lpn | 3.9 | 5.6 | 6.5 | 4.6 | 3.7 | 3.9 | 3.8 | 4.7 |
| 1lpo | 4.2 | 5 | 5.4 | 3.7 | 3.5 | 3.9 | 3.6 | 3.9 |
| 1lpp | 3.6 | 7.7 | 4.5 | 3.2 | 3.8 | 3.6 | 4.2 | 3.8 |
| 1lps | 3.6 | 4.1 | 5 | 5.7 | 4 | 3.7 | 3.7 | 4.7 |
| 3rar | 4.4 | 3.7 | 3.9 | 4.1 | 3.9 | 3.9 | 3.6 | 3.8 |
| 1f6w | 3.6 | 3.9 | 6.6 | 3.5 | 5.6 | 7 | 4.6 | 3.3 |
| 1jmy | 3.7 | 3.5 | 4.1 | 4 | 3.6 | 4.2 | 3.3 | 3.7 |
| 1lpa | 8.9 | 4.4 | 5.2 | 3.8 | 3.6 | 4.2 | 3.6 | 3.8 |
| 3w9u | 4.9 | 3.1 | 3.7 | 3.8 | 4 | 3.7 | 4.1 | 3.6 |
| 4gw3 | 4.1 | 3.9 | 3.7 | 4.1 | 3.5 | 3.9 | 4.5 | 3.5 |
| 4gxn | 2.6 | 3.8 | 4.1 | 3.5 | 3.6 | 4 | 6.9 | 3.8 |
| 6jd9 | 5.7 | 3.4 | 4.4 | 3.6 | 4.1 | 3.6 | 5.1 | 4.2 |
| 2zvd | 4.4 | 4.9 | 5.8 | 3.6 | 3.6 | 3.8 | 4.7 | 5.5 |
| 3a6z | 6.7 | 5.5 | 6.4 | 3.9 | 3.5 | 3.7 | 4.5 | 4.6 |
| 3a70 | 4.2 | 5.2 | 4.6 | 3.4 | 3.7 | 3.6 | 4.6 | 4.3 |
| 6zl7 | 3.5 | 3.9 | 3.7 | 3.4 | 4 | 4.4 | 4.2 | 3.7 |
| 6qin | 3.5 | 3.5 | 4.4 | 3.5 | 5.3 | 3.5 | 3.7 | 4 |
| 6qla | 3.3 | 3.7 | 4.7 | 3.5 | 3.7 | 3.5 | 3.6 | 3.8 |
| 1akn | 3.5 | 3.5 | 4.4 | 3.9 | 3.6 | 3.4 | 3.9 | 7 |
| 1aql | 3.6 | 3.6 | 4.2 | 3.6 | 3.6 | 3.4 | 3.6 | 3.6 |
| 1eth | 3.6 | 3.4 | 3.5 | 2.9 | 3.4 | 3.3 | 3.3 | 3.4 |
| 1ex9 | 3.4 | 3.2 | 4 | 3.7 | 4.3 | 3.5 | 3.7 | 3.8 |
| 1gpl | 3.5 | 4.6 | 3.9 | 3.8 | 3.4 | 3.9 | 3.5 | 3.6 |
| 1k8q | 3.5 | 4.3 | 6.5 | 3.7 | 3.7 | 3.7 | 4.5 | 4 |
| 1llf | 3.4 | 3.9 | 3.6 | 3.2 | 3.3 | 3.3 | 3.4 | 3.3 |
| 2hih | 4.1 | 4.9 | 4.3 | 5.9 | 4.4 | 4.7 | 4.2 | 4.8 |
| 2qua | 3.6 | 4.2 | 5 | 3.6 | 3.6 | 3.6 | 3.6 | 4.3 |
| 2qub | 3.5 | 5 | 5.7 | 4.4 | 3.4 | 3.6 | 5.9 | 4.4 |
| 2w22 | 3.2 | 3.4 | 5.9 | 4.2 | 4 | 4 | 5.7 | 3.8 |
| 3g7n | 4 | 4.8 | 6.6 | 6.3 | 3.6 | 3.5 | 5.4 | 3.7 |
| 5ce5 | 3.6 | 3.9 | 6 | 3.8 | 4.1 | 4 | 5 | 5.6 |
| 5mal | 3.9 | 6.1 | 3.7 | 3.7 | 6.4 | 3.8 | 4.6 | 4.5 |
| 3gbs | 3.5 | 6.9 | 3.9 | 3.6 | 3.9 | 3.7 | 3.7 | 3.7 |
| 3qpd | 4 | 3.8 | 4.2 | 2.1 | 2.4 | 2.2 | 1.5 | 4.4 |
| 5gv5 | 3.6 | 3.6 | 4.4 | 3.9 | 3.7 | 8.9 | 6.9 | 3.6 |
| 4k5q | 4.1 | 4.2 | 4.2 | 3.8 | 3.8 | 3.8 | 7.1 | 7.8 |
| 4k6g | 4.1 | 3.6 | 4.3 | 4.1 | 3.8 | 3.8 | 4.2 | 7.5 |
| 4k6h | 3.6 | 3.8 | 4.3 | 3.7 | 3.9 | 3.9 | 7.8 | 6.3 |
| 4k6k | 9 | 3.8 | 4 | 3.7 | 3.9 | 3.8 | 5.7 | 5.6 |
| 6j1p | 4.1 | 4.4 | 3.7 | 3.7 | 4.1 | 3.6 | 11.9 | 7.6 |
| 6j1r | 3.6 | 3.5 | 3.6 | 3.7 | 4.4 | 3.6 | 5.3 | 3.8 |
| 6j1q | 4.9 | 3.8 | 8.1 | 4.4 | 3.7 | 4.4 | 5 | 4.7 |
| 6j1t | 3.5 | 3.7 | 3.9 | 3.7 | 3.8 | 3.5 | 7.9 | 6.8 |
| 6j1s | 5.1 | 9.3 | 7.1 | 6.2 | 8 | 10.5 | 5.6 | 8.3 |
| 6isq | 6.3 | 1.7 | 3.3 | 1.7 | 1.6 | 1.5 | 9.1 | 8.5 |
| 6isr | 5.1 | 4.1 | 4.3 | 4.9 | 4.1 | 4.2 | 4.9 | 6 |
| 3w9b | 7.5 | 4.2 | 5.9 | 3.9 | 3.6 | 3.7 | 4.2 | 7.4 |
| 4zv7 | 10.3 | 3.8 | 4.5 | 3.8 | 3.8 | 3.7 | 4.9 | 8.9 |
| 6tp8 | 5.9 | 4.6 | 4.5 | 3.6 | 4 | 3.7 | 7.6 | 9.6 |
| 3icv | 3.9 | 6.1 | 4.3 | 4.1 | 3.7 | 4.3 | 3.9 | 3.8 |
| 3icw | 4 | 3.5 | 3.9 | 4.5 | 3.8 | 4.2 | 3.7 | 3.9 |
| 1tcc | 4.4 | 3.6 | 3.8 | 3.6 | 3.6 | 3.7 | 5.4 | 9.1 |
| 1tcb | 4.1 | 3.7 | 4.1 | 3.8 | 3.8 | 3.8 | 7.9 | 6.7 |
| 1tca | 9.3 | 3.9 | 3.6 | 3.5 | 3.8 | 3.7 | 8.4 | 6.9 |
| 1lbt | 4.3 | 3.9 | 3.8 | 3.8 | 4.2 | 3.9 | 8.1 | 8 |
| 1lbs | 4 | 3.5 | 3.3 | 3.6 | 3.5 | 3.7 | 8.1 | 6.1 |
| 6xrv | 3.2 | 4.2 | 5.5 | 3.1 | 3.6 | 3.5 | 3.4 | 5.7 |
| 6xok | 3.4 | 3 | 3.3 | 3.3 | 3.3 | 3.2 | 3.3 | 3.1 |
| 6or3 | 3.2 | 3.9 | 4.1 | 3.6 | 3.1 | 3.2 | 3.3 | 3.3 |
| 5a71 | 3.8 | 4.6 | 4.5 | 3.7 | 3.7 | 3.7 | 8.5 | 5.4 |
| 5a6v | 4.2 | 3.8 | 3.7 | 3.3 | 3.9 | 3.6 | 4.4 | 6.9 |

Notes: The distances between C atoms of ligands at all positions (sn-1, sn-2, or sn-3) and O atom of serine were measured; results listed above were the least value.

Table S3 Distance and Affinity of 6xok with the glycerides of DHA and DHA-ME

| ligand | DHA-TAG | 2-3-DHA-DAG | 2-DHA-DMG | DHA-ME |
| --- | --- | --- | --- | --- |
| Distance(Å) | 3.4 | 3 | 3.3 | 3.3 |
| Affinity(kcal/mol) | -6.6 | -8.3 | -7.3 | -7.9 |


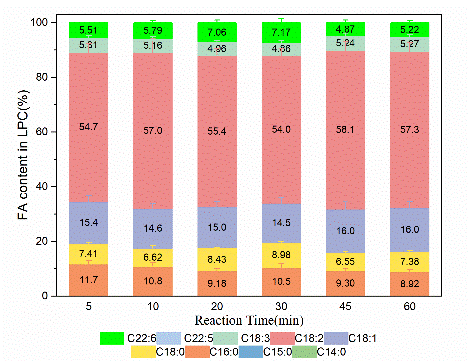

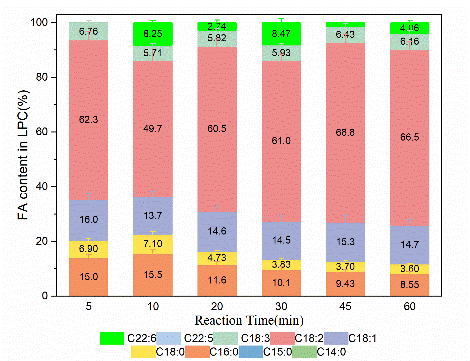

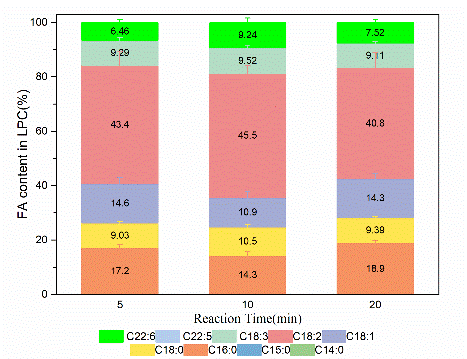


A

B

C

Figure S1 Effect of water content on the PLA1-mediated transesterification--Profiles of FA in the product LPC; A—0.7wt % water in the mixture; B—1.2wt % water in the mixture; C—1.7wt % water in the mixture; Reaction conditions: 20 mg PC, 1.0 g algae oil, 10 µL PLA1, 45 ^o^C, 500rpm. Notes: water herein refers to phosphate buffer solution (PBS,0.1M, pH 7.5).





Figure S2 Profiles of FA in the product LPC at different temperature; Reaction conditions: 20 mg PC, 1.0 g algae oil, 10 µL PLA1, 500rpm, 30min.





Figure S3 Profiles of FA in the product LPC in PLA1-mediated transesterification of PC with DHA-ME; Reaction conditions: 20 mg PC, 1.0 g DHA-ME derivatized from algae oil, 10 µL PLA1, 45 ^o^C, 500rpm.





Figure S4 Profiles of FA in the product LPS in PLA1-mediated transesterification of PS with DHA-TAG; Reaction conditions: 20 mg PS, 1.0 g algae oil, 10 µL PLA1, 45 ^o^C, 500rpm.


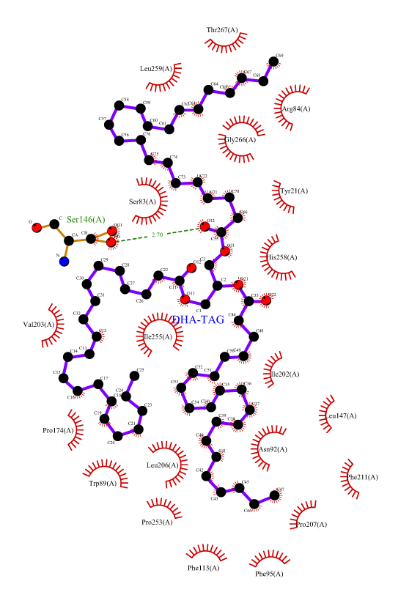

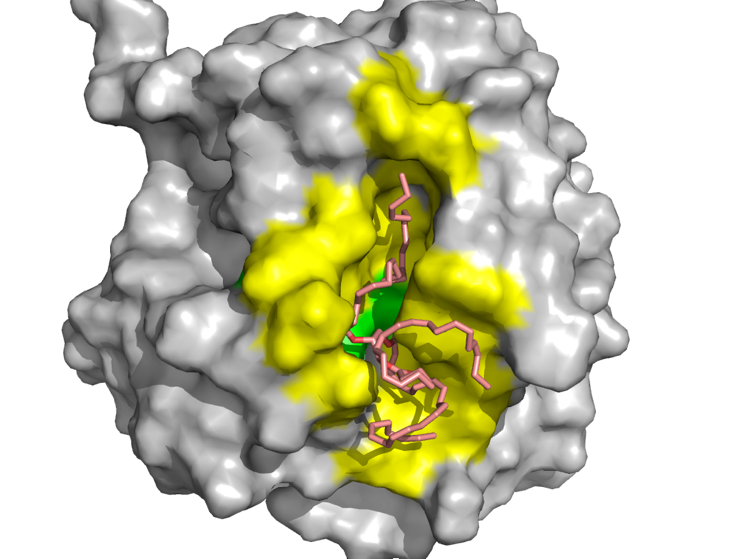


A

B

Figure S5 Visual results of interaction between DHA-TAG and 6xok, generated by molecular docking, were shown by (A) 2D; and (B) 3D models; the active pocket of 6xok was shown in yellow and the catalytic triad was shown in green.

A

B


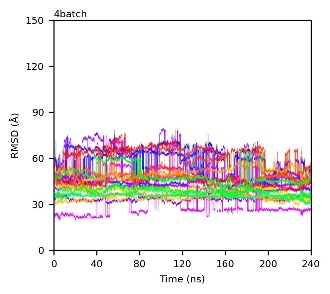

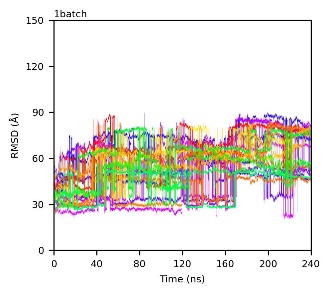

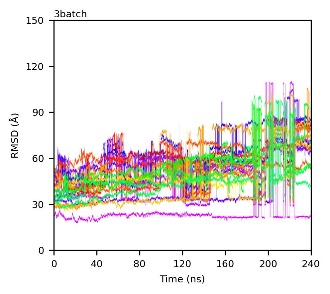

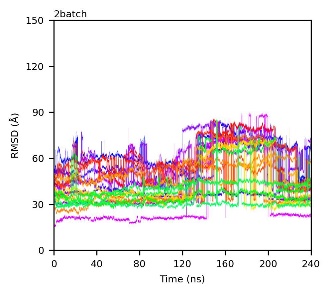

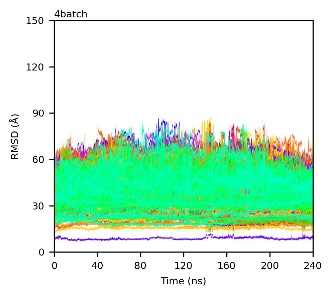

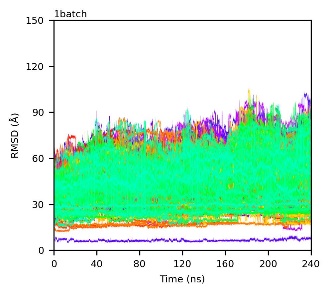

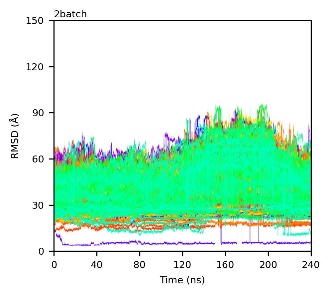

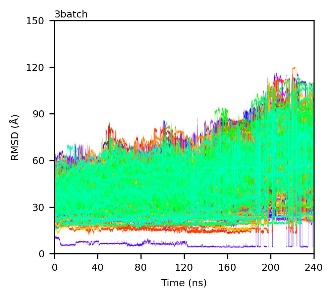


Figure S6 Time evolution of RMSD for the distance of acyl C atom of (A) PC; (B) DHA-TAG to O atom (Ser146) in solvent-free system without water during 4 times of MD simulations. In each case, all 20 PC molecules and 200 DHA-TAG molecules were analyzed. The sudden change in the value of RMSD was caused by periodic boundary conditions, in which molecules entered the next reaction micro-unit and mirrored in the opposite location. It’s a common phenomenon in MD simulations and didn’t have any negative on the whole simulation process.


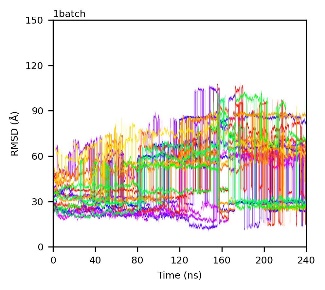

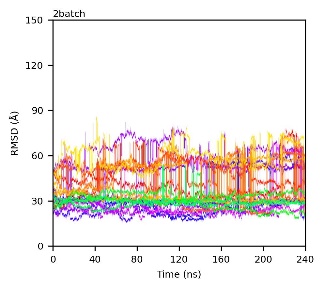

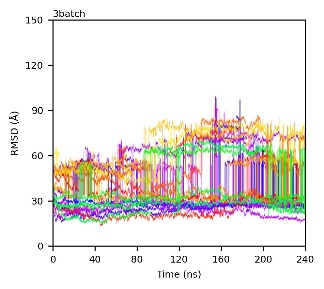

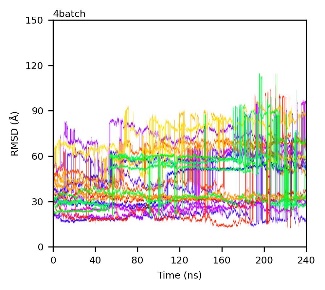

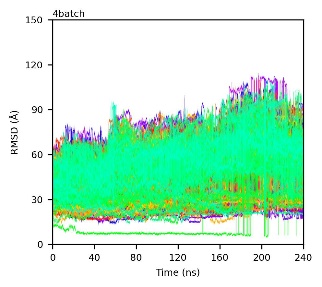

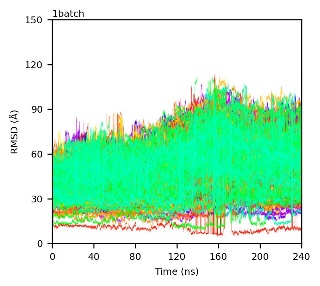

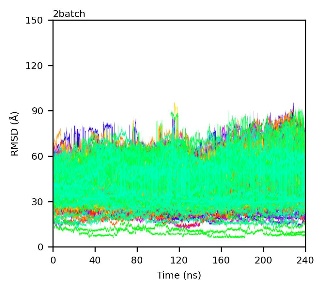

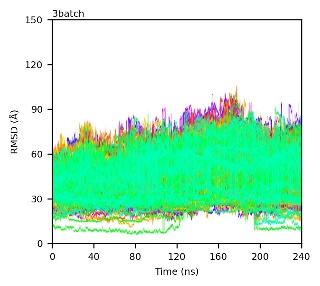


A

B

Figure S7 Time evolution of RMSD for the distance of acyl C atom of(A) PC; (B) DHA-TAG to O atom (Ser146，-OH) in solvent-free system with micro-water during 4 times of MD simulations. In each case, all 20 PC molecules, 200 DHA-TAG molecules, and 770 water molecules were analyzed. The sudden change in the value of RMSD was caused by periodic boundary conditions, in which molecules entered the next reaction micro-unit and mirrored in the opposite location. It’s a common phenomenon in MD simulations and didn’t have any negative on the whole simulation process.


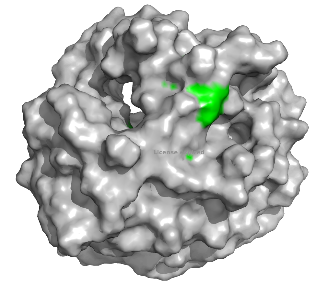

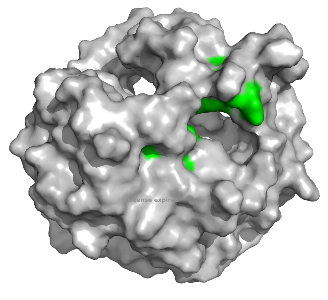

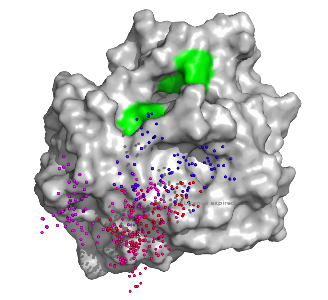

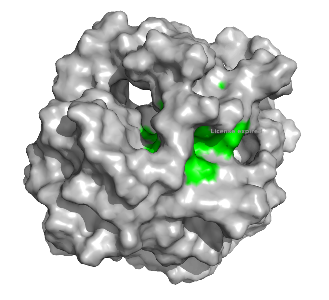

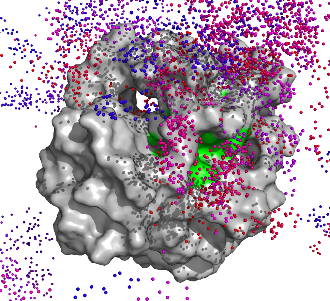

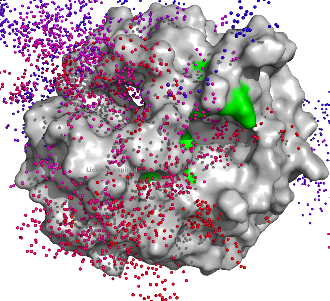

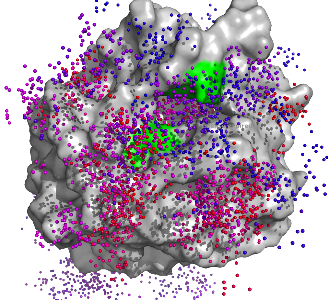

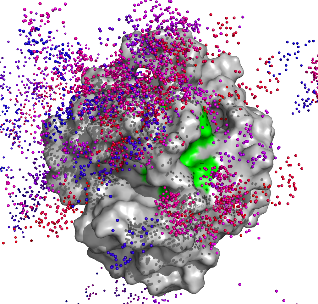


A

B

1batch

2batch

3batch

4batch

1batch

2batch

3batch

4batch


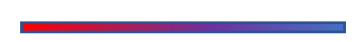


0 240ns

Figure S8 Trajectories of the substrates (A) PC; (B) DHA-TAG in solvent-free system without water under 4 MD simulation; Only trajectories of the substrates which could enter the active pocket were shown. Other molecules were hidden for the easier visualization. The catalytic triad residues (S146-D201-H258) were represented as green color to guide the location of the active pocket.


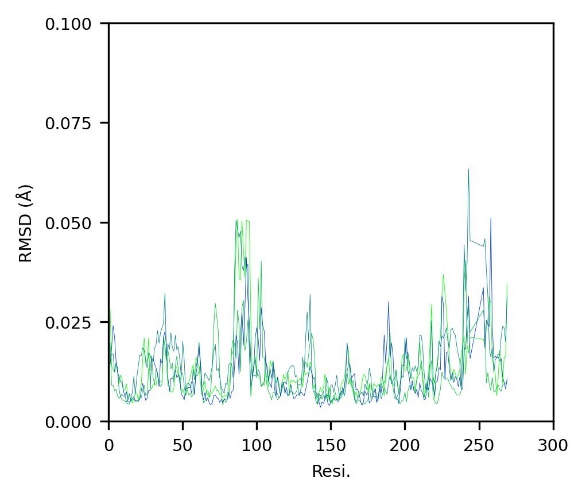

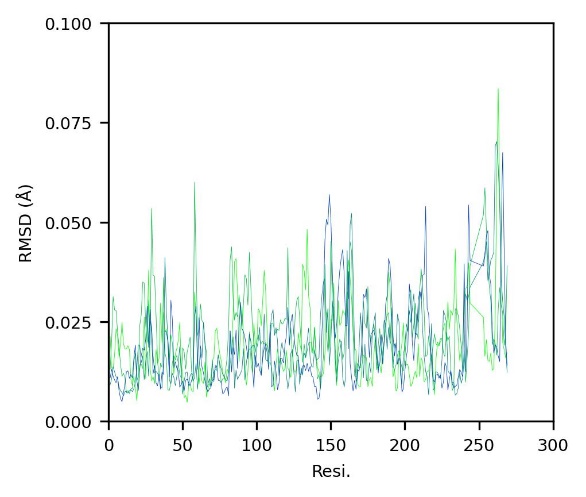


A

B

Figure S9 RMSD of each residue of PLA1 in (A) anhydrous system, and (B) micro-water system.


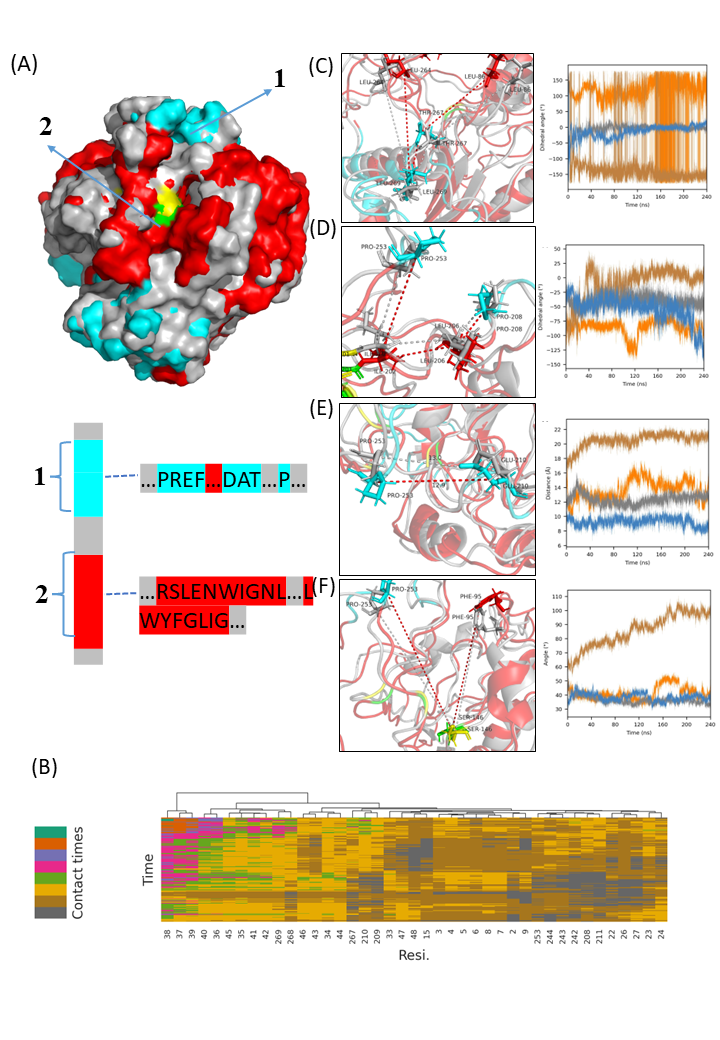


Figure S10 MD results for the anhydrous system; (A) Comparison of the two protein molecular conformations of the original PLA1 (gray) and the PLA1 in the process of diffusion (red and cyan); the marked regions (1,2; amino acid sequences have been given) show the significant differences between the two PLA1 conformations and the cyan regions represent the better affinities with PC and DHA-TAG according to the results of (B); (B) Statistics of average interaction frequencies of each residue with substrate molecule DHA-TAG and PC; The cutoff value was used to highlight the residues which have stronger affinities with substrates, which were set as 70. If the total interaction frequency of a residue with substrate during the whole simulation time was less than the cutoff value, this residue was hidden for the easier visualization. The interaction distance was set as 8 Å due to a larger steric hindrance; (C) Time evolution of the dihedral angle among L264, L269, T267, and L86 and its visualization result; (D) Time evolution of the dihedral angle among P253, I202, L206, and R205 and its visualization result; (E) Time evolution of the distance between P253 and E210 and its visualization result; (F) Time evolution of the angle among P253, S146 and F95 and its visualization result.
